# Consumer Artificial Intelligence for Pedigree Analysis: A Comparative Study in Medical Genetics

**DOI:** 10.7759/cureus.117953

**Published:** 2026-10-15

**Authors:** Seba S Abduljabbar, Mohammed Alqarni, Alawi Habara

**Affiliations:** 1 Medicine, Imam Abdulrahman Bin Faisal University, Dammam, SAU; 2 Biochemistry, Imam Abdulrahman Bin Faisal University, Dammam, SAU

**Keywords:** artificial intelligence, chatgpt, claude, gemini, large language models, medical education, medical genetics, pedigree analysis

## Abstract

Background: Large language models (LLMs) are increasingly used in medical education, but their ability to analyze pedigree-based genetics problems is not well defined. This exploratory study aimed to compare the accuracy of four consumer LLMs on image-based pedigree questions and to describe the sources of their errors.

Methods: Nine pedigree cases comprising 19 sub-questions were submitted as images on May 18, 2026, to the free consumer versions of ChatGPT (GPT-5.5), Gemini (Gemini 3 Flash), Claude (Claude Sonnet 4.6), and the Microsoft Copilot app, once each in a new chat, without retries or follow-up prompts. The primary measure was accuracy, defined as the number and percentage of sub-questions answered correctly against a predefined answer key reviewed by a medical geneticist. Item-level scores were re-checked during revision using uniform rules. Incorrect answers were retrospectively classified as perception/structure, reasoning/knowledge, mixed or indeterminate errors, or as reported image-access failures.

Results: ChatGPT and Claude each answered 14 of 19 sub-questions correctly (73.7%), Copilot 11 (57.9%), and Gemini three (15.8%). Twelve of Gemini's 16 incorrect answers followed its report that it could not see or load the image. Claude's errors in the double-cousin pedigrees reflected misreading of the mating lines, whereas ChatGPT's mainly involved assigning the degree of relationship. In the single atypical pedigree (dominantly inherited hemoglobin S-Oman), no model identified the variant; Claude described dominant features but attributed them to pseudodominance because sickle cell disease is recessive.

Conclusions: In this small, single-attempt task set without statistical comparison, consumer LLMs showed variable accuracy, and errors arose both from misreading pedigrees and from misapplying genetic principles. These results do not establish a ranking of the models or an educational benefit. Consumer AI assistants should complement, not replace, formal instruction and expert supervision in medical genetics.

## Introduction

Medical education is being revolutionized through AI. In theory, AI will be an integral part of medical education, serving as a collection of educational tools that provide feedback, simulate, analyze data, and develop curricula. AI is especially important in high-volume, high-stakes competency-based training environments where educators are trying to teach large amounts of content in a very short amount of time [1-3]. Several recent surveys show that this is a discipline that has transitioned from experimental to organizational in terms of adoption, especially in undergraduate and pre-clinical education, where educators are looking for ways to make learning more personalized, more digital, and more adaptable to individual needs. Scoping reviews and narrative reviews both showed that AI was heavily utilized in adaptive learning, clinical reasoning support, automated assessment, and virtual patients; similarly, both types of reviews also found that there was considerable variability across institutions and geographies regarding the use of AI in medical education [1,4-6]. The variability in the implementation of AI across institutions and geographies is critical because, by the time future physicians enter their first clinical setting, they will likely have experience using algorithmic aids for documentation, diagnosis, and decision-making. Thus, medical schools have a dual responsibility to teach students to learn using AI when it helps them learn and to teach students about AI so that students understand the potential for misuse and the limitations of AI. However, AI's application in medical education is constrained by current ethical, professional, and academic concerns. Overall, studies demonstrate that both students and educators hold favorable views of AI while expressing fears about privacy, discrimination, over-reliance, lack of transparency, cheating, and inequality in the availability of training and resources [3,4,7]. Although there are no perfect solutions to these problems, studies of chatbots have found that they have been used successfully to enhance bedside teaching and the clinical history-taking process, but were less successful in assessing biology, image-based assessment, and critical thinking [8,9]. Large language model (LLM)-based chatbots are conversational artificial intelligence systems that have become increasingly integrated into medical education. They can explain concepts, summarize content, generate practice questions, provide rapid feedback, and support self-directed learning, making them valuable educational tools [1,2,5]. However, their effectiveness depends on appropriate task selection and academic supervision, and independent evaluation remains essential before they can be relied upon for specialized educational tasks, such as pedigree interpretation [8,9].

ChatGPT (OpenAI, San Francisco, CA) is a multimodal LLM capable of processing both text and image inputs. In this study, ChatGPT was evaluated through its free consumer interface using GPT-5.5 [10-13]. Owing to its widespread use in medical education and ability to generate explanations and educational content, ChatGPT has become one of the most commonly used AI assistants for learning [9,14]. Gemini 3 Flash (Google DeepMind, Alphabet Inc., Mountain View, CA) is a multimodal LLM capable of processing text, images, audio, video, and code [15]. During the study period, the free version was evaluated because of its wide accessibility and multimodal capabilities, which support educational tasks such as concept explanation, case analysis, and interpretation of image-based materials. According to Google, Gemini 3 Flash is designed to balance reasoning performance with speed [14,15]. Copilot (Microsoft® Corp., Redmond, WA) is a conversational AI assistant developed by Microsoft to assist with searching, writing, content generation, and information retrieval [16-18]. The consumer Copilot app evaluated in this study is distinct from Microsoft 365 Copilot, the workplace product that is integrated with applications such as Word, Excel, PowerPoint, Outlook, and Teams [16,19]; these integrations were not evaluated. Claude (Anthropic PBC, San Francisco, CA) is a multimodal family of LLMs designed for reasoning, writing, coding, and information synthesis [20]. It can process both text and images, making it suitable for educational tasks requiring multimodal interpretation [21]. The free consumer version, which used Claude Sonnet 4.6, was evaluated in this study [22]. One area in which the educational performance of LLMs remains insufficiently studied is pedigree interpretation. Pedigree analysis requires learners to integrate family history, inheritance patterns, and genetic principles and accurately interpret graphical family trees and answer associated clinical questions. Because these tasks combine visual interpretation with analytical reasoning, evaluating the performance of current LLMs in this educational context is important.

The primary objective of this study was to compare the accuracy of four consumer LLMs (ChatGPT, Gemini, Copilot, and Claude) in answering image-based pedigree interpretation questions when each question set was submitted once without follow-up prompting. Accuracy was defined as the number and percentage of sub-questions answered correctly according to a predefined answer key. The secondary objectives were to describe the main errors made by each model and to retrospectively classify incorrect answers according to whether they arose from misreading the pedigree (perception/structure errors) or from applying incorrect genetic principles or knowledge to a correctly read pedigree (reasoning/knowledge errors). No hypothesis was established before testing; the study was designed as an exploratory, descriptive comparison intended to generate rather than test hypotheses.

## Materials and methods

Study design and pedigree cases

This study compared the performance of four consumer LLMs - ChatGPT, Gemini, Copilot, and Claude - on pedigree-based medical genetics questions. A total of nine pedigree cases were included, with two to three related questions for each pedigree. The prompts and answer key contained 19 separately numbered sub-questions (two each for Pedigrees 1-6, one for Pedigree 7, and three each for Pedigrees 8 and 9), and each sub-question was scored separately. The tasks assessed inheritance pattern recognition, genotype inference, risk estimation, degree of relationship, DNA sharing, and, in one pedigree, diagnosis. The study was exploratory and descriptive, and no statistical hypothesis testing was performed.

Search strategy

A literature search was conducted using the following terms: ("large language model*" OR "generative artificial intelligence") AND ("medical genetics" OR genetic* OR inheritance OR pedigree*) AND (performance OR accuracy OR "diagnostic accuracy"), specifically targeting the title and abstract fields. Additionally, the official webpages of OpenAI (ChatGPT), Google/Google DeepMind (Gemini), Microsoft (Copilot), and Anthropic (Claude) were used to extract background information specific to each AI tool.

LLM testing procedure

All pedigrees were presented as images to preserve the visual nature of pedigree analysis. Each pedigree image and its accompanying question(s) were tested once using the official consumer version of each LLM. No retries, rewording, follow-up questions, or regenerated responses were allowed. Responses were recorded exactly as generated, and failure to interpret the pedigree image or provide a usable answer was scored as incorrect.

The exact text submitted with each pedigree image, including the answer options, is reproduced verbatim in the "Researcher Prompt" panel of the corresponding supplementary figure, alongside the unedited model response (Appendix 1, Supplementary Figures S1-S18). The prompts consisted of the question text and answer options shown in these panels. The questions and answer options were identical across the four platforms, apart from minor formatting differences and the presence or absence of the introductory line "The following questions are related to the pedigree."

All testing was conducted on May 18, 2026. Each assistant was accessed through its free consumer interface with default settings: ChatGPT using GPT-5.5 [10-13], Gemini using Gemini 3 Flash [15], Claude using Claude Sonnet 4.6 [22], and the consumer Microsoft Copilot app [17,18], whose underlying model was not documented. Microsoft 365 Copilot and its integrations with Word, Excel, and other applications were not evaluated. Each pedigree, with its associated questions, was submitted in a new chat.

Response evaluation

Responses were compared with a predefined answer key. Performance was summarized at the pedigree level by recording the number of correctly answered sub-questions for each LLM, together with brief notes on major errors. The pedigree cases, associated questions, and answer key are provided in Appendix 2 (Supplementary Figures S19-S22).

Performance measures and scoring rules

The primary performance measure was overall accuracy, defined as the number of sub-questions answered correctly out of 19, expressed as a percentage. The secondary measures were (i) the pedigree-level score, defined as the number of correctly answered sub-questions for each pedigree; (ii) reported image-access failure, recorded when a model stated that it could not see, load, or read the pedigree image or asked the user to describe it; and (iii) the source of each error, classified as described below. The quality of explanations was described qualitatively and separately from final-answer accuracy, and correct final answers accompanied by flawed reasoning were identified.

The original protocol specified that responses were compared with the predefined answer key and that failure to interpret the pedigree image or to provide a usable answer was scored as incorrect. Detailed rules for other response types were not recorded at the time of testing. During revision, because the originally reported totals did not equal the sums of the pedigree-level scores, all item-level scores were re-checked against the recorded responses (Appendix 1, Supplementary Figures S1-S18) and the answer key (Appendix 2, Supplementary Figures S19-S22) using the following rules, applied identically to all four models. Each sub-question was scored as correct or incorrect, with no partial credit. For items with answer options, or requesting a single value or genotype, the final answer stated by the model was compared with the key, and a correct final answer was scored as correct even when the accompanying explanation was flawed. Open-ended explanation items were scored as correct only when the explanation contained the key concept specified in the answer key (genetic complementation for Pedigree 5, autosomal dominant inheritance with reduced penetrance for Pedigree 7, and the dominant sickle syndrome HbS-Oman for Pedigree 9); a partially correct explanation that omitted this concept was scored as incorrect. When a response contained internally inconsistent statements, the final answer explicitly stated was scored, and an item without an identifiable final answer was scored as incorrect. An answer given for a different individual from the one asked about was scored as incorrect. Consistent with the original protocol, when a model stated that it could not see, load, or read the pedigree image, all sub-questions for that pedigree were scored as incorrect, including answers offered from the question text alone.

This re-check changed two item scores and corrected three totals. Copilot's answer to Pedigree 1, question 1 had been scored as correct, but the recorded response selected autosomal recessive rather than X-linked recessive inheritance (Appendix 1, Supplementary Figure S10); it was rescored as incorrect. Claude’s explanation for Pedigree 9, question 3 had been scored as correct, although it attributed the pattern to pseudodominance of an autosomal recessive disorder and did not identify a dominant sickle syndrome (Appendix 2, Supplementary Figure S18); it was rescored as incorrect, consistent with the scoring of the corresponding explanations of ChatGPT and Copilot. In addition, the totals previously reported for ChatGPT (15) and Copilot (13) did not equal the sums of their pedigree-level scores (14 and 12, respectively). The corrected totals are 14 of 19 for ChatGPT, 14 for Claude, 11 for Copilot, and three for Gemini (unchanged). Because of the small number of items and the single attempt per model, results are reported descriptively, and no statistical comparisons between models were performed.

Answer-key validation

The answer key was prepared before testing and specifies the correct option or key concept for each sub-question (Appendix 2, Supplementary Figures S19-22). The predefined answer key was reviewed for accuracy by Dr Alawi Habara, a medical geneticist and coauthor of this study. Because the reviewer is a coauthor, this review was not independent, and the key was not validated by additional geneticists. For Pedigrees 3 and 4, the key defines the degree of relationship genealogically, so first cousins are third-degree relatives even when, as double first cousins, they share more DNA than ordinary first cousins.

For Pedigree 9, the key specifies sickle cell disease (SCD) as the most likely diagnosis, autosomal dominant inheritance as the most likely mode of inheritance and, as the expected explanation, hemoglobin S-Oman (HbS-Oman), a dominant sickle syndrome in which two mutations on a single β-globin allele cause sickling in heterozygotes despite the presence of normal adult hemoglobin (HbA) (Appendix 2, Supplementary Figure S22) [23]. These answers differ in how far they can be established from the pedigree. The mode of inheritance can be inferred from the pedigree itself: affected individuals appear in each generation, affected × unaffected matings produce affected offspring of both sexes, and male-to-male transmission (I.1-II.5 and II.5-III.4) excludes X-linked inheritance. Autosomal recessive inheritance with pseudodominance cannot be excluded from the pedigree alone, but it would require all three unaffected individuals who married into the affected line (I.2, II.4, and II.7) to be carriers, which is less likely; the question therefore asked for the most likely mode of inheritance. The diagnosis of SCD rests on the clinical description in the question stem rather than on the pedigree. HbS-Oman is the expected explanation for the combination of a sickling disorder and dominant transmission, based on its published description [23], but the specific variant cannot be established from a pedigree; in a real family, hemoglobin analysis or β-globin gene testing would be required.

Retrospective error classification

Because the original scoring recorded only whether each answer was correct, each sub-question scored as incorrect was retrospectively classified during revision, using only the content of the recorded responses (Appendix 1, Supplementary Figures S1-S18). A perception/structure error was assigned when the response misidentified pedigree symbols, shading, mating lines, twin symbols, or relationships. A reasoning/knowledge error was assigned when the response described the relevant pedigree features without evident misreading but applied an incorrect genetic principle or did not apply relevant genetic knowledge. A mixed error was assigned when observable perception and reasoning mistakes both contributed. An error was classified as indeterminate when the response did not provide enough evidence to identify its source, for example, when only an answer option was given. Statements by a model that it could not see, load, or read the image were recorded separately as reported image-access failures; their technical cause could not be determined from the responses.

The classification was based only on what each response explicitly stated and does not imply knowledge of the internal processes of the models; an incorrect answer was not assumed to reflect a perception error. Correct final answers accompanied by flawed reasoning were not rescored but are identified separately. The classification was performed retrospectively without blinded or independent raters, and no agreement statistics were calculated; it is therefore descriptive and does not alter the accuracy scores.

## Results

Across 19 sub-questions derived from nine pedigree cases, ChatGPT and Claude each answered 14 correctly (73.7%), Copilot answered 11 correctly (57.9%), and Gemini answered three correctly (15.8%) (Table 1). These totals incorporate the item-level re-check described in the Materials & Methods and equal the sums of the pedigree-level scores in Table 2. The complete AI outputs corresponding to the evaluated pedigree cases are provided in the Appendices (Supplementary Figures S1-18).

**Table 1:** Overall scores, accuracy, and common weaknesses of ChatGPT, Gemini, Copilot, and Claude.

AI assistant	Total correct answers out of 19	Accuracy (%)	Most common weakness
ChatGPT	14	73.7%	Fail to handle special cases that require a deep search
Gemini	3	15.8%	Reported inability to see, load or read the pedigree image
Copilot	11	57.9%	Fail to recognize the details of a pedigree (such as a double cousin)
Claude	14	73.7%	Fail to interpret complex pedigree structures, resulting in confident but incorrect reasoning

**Table 2:** Pedigree-level scores, key errors, and retrospective error-source classification for each AI assistant. * The model reported that it could not see, load, or read the pedigree image; all sub-questions for that pedigree were scored as incorrect. In Pedigree 9, Gemini's text-based answer to question 1 (sickle cell disease) matched the key but was scored as incorrect under this rule. † Retrospective classification of sub-questions scored as incorrect (see Materials & Methods; details in Table 3). ‡ Indeterminate because the pedigree image received by ChatGPT for Pedigree 9 could not be verified (Appendix 1, Supplementary Figure S5). Scores for Copilot (Pedigree 1) and Claude (Pedigree 9) reflect the item-level re-check described in the Materials & Methods. Recorded responses: ChatGPT (Supplementary Figures S1-S5); Gemini (Supplementary Figures S6-S9); Copilot (Supplementary Figures S10-S14); Claude (Supplementary Figures S15-S18; answer key (Supplementary Figures S19-S22). P, perception/structure error; R, reasoning/knowledge error; I, indeterminate; IA, reported image-access failure; no error met the criteria for a mixed error; Q, sub-question

Pedigree	Main Concept Tested	No. of Questions	Gemini Score	Copilot Score	Claude Score	ChatGPT Score	Error Source (Retrospective)†	Key Error/Comment
1	X-linked recessive	2	0*	1	2	2	Gemini Q1-2: IA; Copilot Q1: I	ChatGPT and Claude answered both questions correctly with step-by-step genetic reasoning. Copilot gave answer-only responses and selected autosomal recessive rather than X-linked recessive inheritance, although its 100% carrier probability for II.6 was correct. Gemini reported that no image had been provided.
2	Mitochondrial inheritance	2	1	0*	2	2	Gemini Q1: R; Copilot Q1-2: IA	ChatGPT and Claude answered both questions correctly. Gemini read the pedigree but selected autosomal dominant inheritance; its 0% answer was correct but was reached through autosomal dominant reasoning. Copilot reported that it could not reliably read which individuals were affected.
3	DNA shared among relatives	2	0*	1	0	1	Gemini Q1-2: IA; Copilot Q1: I; Claude Q1-2: P; ChatGPT Q2: R	The pedigree shows a double first-cousin pattern (the individuals in the third generation are first cousins through both their fathers and their mothers), so they share 25% of their DNA and are third-degree relatives under the answer key. ChatGPT identified the double-cousin structure and gave 25% but classified the relationship as second degree. Copilot’s answer-only response gave 12.5%, the value for ordinary first cousins, and correctly gave third degree. Claude identified the individuals as full siblings (50% DNA shared and first-degree relatives). Gemini reported that it needed to see the pedigree chart.
4	DNA shared among relatives	2	0*	1	1	0	Gemini Q1-2: IA; Copilot Q2: P; Claude Q2: P; ChatGPT Q1: P, Q2: R	The pedigree shows a double-cousin pattern from parents who are identical twins, so the individuals in the third generation share 50% of their DNA even though they are first cousins (third-degree relatives). Copilot and Claude both selected 50% but did so by reading III.1 and III.2 as full siblings, and both therefore answered first degree. ChatGPT identified the double-cousin structure but not the twin relationship of the parents and therefore answered 25%. Gemini reported that the file appeared empty.
5	Genotype interference/autosomal recessive	2	2	2	2	2	None	All four assistants explained the result by genetic complementation and gave a 100% carrier probability.
6	Autosomal dominant	2	0*	2	2	2	Gemini Q1-2: IA	ChatGPT, Claude and Copilot answered both questions correctly; in the authors’ assessment, ChatGPT’s explanation was easier to follow, whereas Claude’s tone was more formal. Gemini reported that the image had not been uploaded or processed.
7	Autosomal dominant with penetrance	1	0*	1	1	1	Gemini Q1: IA	ChatGPT, Claude and Copilot identified autosomal dominant inheritance with reduced penetrance; Claude gave the most detailed justification. Gemini reported that the image was blank and gave general guidance only.
8	X-linked dominant	3	0	1	3	3	Gemini Q1-3: R; Copilot Q1: I, Q3: I	Copilot and Gemini selected autosomal dominant rather than X-linked dominant inheritance. For the third question, Copilot answered for II.6 rather than II.8. Gemini read the pedigree, including the affected father’s affected daughters, but answered all three questions incorrectly.
9	Dominant sickle syndrome (special)	3	0*	2	1	1	Gemini Q1-3: IA; Copilot Q3: R; Claude Q2-3: R; ChatGPT Q2-3: I‡	The pedigree shows a sickle cell disease phenotype with autosomal dominant transmission, consistent with the rare dominant sickle syndrome HbS-Oman. No assistant identified HbS-Oman. Copilot identified autosomal dominant inheritance; ChatGPT and Claude selected autosomal recessive inheritance; Gemini reported that the image did not load and answered from the clinical description.
Total	All pedigrees	19	3	11	14	14	-	Totals equal the overall scores in Table 1.

**Table 3:** Retrospective classification of incorrect answers and correct final answers reached with flawed reasoning. Classes: P, perception/structure error; R, reasoning/knowledge error; I, indeterminate; IA, reported image-access failure. P, pedigree; Q, sub-question Figure numbers refer to the supplementary figures of recorded responses. ‡ Indeterminate because the pedigree image received by ChatGPT for Pedigree 9 could not be verified.

Model	Pedigree, Item	Answer Given (Key)	Class	Observable Mistake (Figure)
Incorrect answers				
ChatGPT	P3 Q2	Second degree (third degree)	R	Identified III.1 and III.2 as double first cousins but assigned the degree of relationship from expected DNA sharing (25%) rather than from the genealogical relationship (2-A).
ChatGPT	P4 Q1	25% (50%)	P	Described II.1/II.2 and II.3/II.4 as ordinary sibling pairs; the twin symbols were not acknowledged, so the twin-derived 50% sharing was missed (2-B).
ChatGPT	P4 Q2	Second degree (third degree)	R	Assigned the degree of relationship from expected DNA sharing, as in Pedigree 3 (2-B).
ChatGPT	P9 Q2-3	Autosomal recessive; recessive explanation without HbS-Oman (autosomal dominant; HbS-Oman)	I‡	Stated that sickle cell disease “is inherited in an autosomal recessive pattern” and that “the pedigree perfectly matches”; the image received could not be verified (5).
Claude	P3 Q1-2	50%; first degree (25%; third degree)	P	Read III.1 and III.2 as full siblings, “both children of II.2 and II.3” (15-C).
Claude	P4 Q2	First degree (third degree)	P	Stated that the lines to II.1 and II.4 show siblings, “not additional parents”, and treated III.1 and III.2 as full siblings (16-A).
Claude	P9 Q2-3	Autosomal recessive; pseudodominance explanation (autosomal dominant; HbS-Oman)	R	Described dominant-appearing features and male-to-male transmission but attributed them to pseudodominance from assumed carrier spouses because sickle cell disease is recessive; HbS-Oman not considered (18-B).
Copilot	P1 Q1	Autosomal recessive (X-linked recessive)	I	Answer-only response; no reasoning shown (10-A).
Copilot	P2 Q1-2	No answer (mitochondrial; 0%)	IA	Stated that it could not reliably read which individuals were affected and asked the user to list them (10-B).
Copilot	P3 Q1	12.5% (25%)	I	Answer-only; the value corresponds to ordinary first cousins, but the response does not show whether the structure was misread (11-A).
Copilot	P4 Q2	First degree (third degree)	P	Stated that III.1 and III.2 “are the two children of the same parents (II.2 and II.3)” (11-B).
Copilot	P8 Q1	Autosomal dominant (X-linked dominant)	I	Answer-only response (13-B).
Copilot	P8 Q3	Second degree, for II.6 (unrelated, for II.8)	I	Answered for II.6 instead of the queried individual II.8; the source of the substitution is not evident (13-B).
Copilot	P9 Q3	Dominant explanation without HbS-Oman (HbS-Oman)	R	Identified vertical and male-to-male transmission but did not explain how a sickling disorder could be dominantly inherited (14).
Gemini	P1, P3, P4, P6, P7, P9 (12 items)	No answer or text-based answer (various)	IA	Stated that no image had been provided, that the file appeared empty or blank, that the image was not uploaded or processed, or that it did not load (6-A, 6-C, 7-A, 8-A, 8-B, 9-B).
Gemini	P2 Q1	Autosomal dominant (mitochondrial)	R	Noted affected mother I.2 with affected sons and daughters and affected fathers II.3 and II.6 with unaffected children, but did not consider exclusive maternal transmission (6-B).
Gemini	P8 Q1	Autosomal dominant (X-linked dominant)	R	Noted that affected I.1 transmitted the trait to all his daughters but chose autosomal dominant as the “simplest” explanation (9-A).
Gemini	P8 Q2	Third degree (second degree)	R	Traced III.1 to II.7 correctly but classified an aunt as a third-degree relative by counting three steps (9-A).
Gemini	P8 Q3	Third degree (unrelated)	R	Recognized II.8 as the spouse of II.7 but classified a relative by marriage (“uncle-in-law”) as a third-degree relative (9-A).
Correct final answers with flawed reasoning (scored as correct)				
Copilot	P4 Q1	50% (50%)	-	Reached 50% by treating III.1 and III.2 as full siblings (11-B).
Claude	P4 Q1	50% (50%)	-	Reached 50% by treating III.1 and III.2 as full siblings (16-A).
Gemini	P2 Q2	0% (0%)	-	Reached 0% by reasoning that unaffected individuals in an autosomal dominant disorder are not carriers (6-B).

ChatGPT and Claude showed similar performance across standard pedigree interpretation tasks and, in the authors' qualitative assessment, generally provided more detailed explanations than the other assistants (Table 2). Although both chatbots obtained the same overall score, ChatGPT correctly read the double-cousin structure in Pedigree 3, whereas Claude misread the relationship in both double-cousin pedigrees (Table 2). Copilot performed reasonably well on conventional inheritance questions but showed weakness in recognizing more complex pedigree relationships, particularly double-cousin structures. Gemini obtained the lowest score, largely because, in six of the nine pedigrees, it reported that it could not see, load, or read the image; these pedigrees were scored as incorrect under the study design. Because each model answered each question set once and no statistical testing was performed, these differences describe performance on this task set and do not establish a ranking of the models.

At the pedigree level, ChatGPT and Claude each achieved full scores in six of the nine pedigrees and obtained the highest or joint-highest score in most pedigrees. Despite the fact that both obtained the exact scores, their AI styles in answering the pedigrees differed. ChatGPT's errors were confined to the double-cousin pedigrees and the dominant sickle syndrome pedigree, whereas Claude provided extended explanations but reached incorrect conclusions in the double-cousin pedigrees because of structural misinterpretation, and in the dominant sickle syndrome pedigree. Copilot matched ChatGPT in several standard inheritance cases but lost accuracy in pedigrees requiring careful recognition of relationship structure or more detailed reasoning. All four assistants struggled with the special pedigree involving the dominant sickle syndrome scenario, and in this single case even the higher-scoring systems did not identify the atypical inheritance pattern. The screenshots of the answers for each Chatbot are in the supplementary material.

Of the 34 incorrect answers across the four models, 14 were reported image-access failures (12 for Gemini and two for Copilot), five were perception/structure errors, nine were reasoning/knowledge errors, and six were indeterminate; none met the criteria for a mixed error (Tables 2-3). In six of the nine pedigrees, Gemini stated that no image had been provided, that the file appeared empty or blank, or that the image did not load (Supplementary Figures S6-S9). Its four other errors were reasoning/knowledge errors in pedigrees that it described without evident misreading; for example, in Pedigree 8 it noted that the affected father transmitted the trait to all his daughters but selected autosomal dominant inheritance (Supplementary Figure S9). Claude's errors in Pedigrees 3 and 4 were perception/structure errors, because it described III.1 and III.2 as full siblings born to II.2 and II.3 (Supplementary Figures S15-S16); its two Pedigree 9 errors were reasoning/knowledge errors. ChatGPT identified the double-cousin structure in Pedigree 3 but assigned the second degree of relationship on the basis of expected DNA sharing (a reasoning/knowledge error), and in Pedigree 4, it did not acknowledge the twin symbols (a perception/structure error) (Supplementary Figure S2). Four of Copilot's eight errors were indeterminate because its responses gave answers without explanations (Supplementary Figures S10-S14). Three correct final answers were accompanied by flawed reasoning: Copilot and Claude both selected 50% for Pedigree 4 after describing III.1 and III.2 as full siblings, and Gemini gave the correct carrier probability (0%) for Pedigree 2 after concluding autosomal dominant inheritance (Table 3).

In the supplementary record, Supplementary Figure S5 displays the Pedigree 7 image alongside ChatGPT's response to the Pedigree 9 questions. The response refers to individuals who appear only in Pedigree 9 (II.6, II.7, and III.4-III.6), but the archived materials do not establish which image ChatGPT received. ChatGPT's Pedigree 9 answers were scored against the Pedigree 9 answer key, which does not depend on the image received, and the sources of its two errors were classified as indeterminate.

In Pedigree 9 (HbS-Oman), all four models selected SCD as the most likely diagnosis, but no response mentioned HbS-Oman, and the responses diverged on the mode of inheritance (Supplementary Figures S5, S9, S14, and S18). Claude described dominant-appearing features, stating that "at first glance the pedigree looks like it could be dominant" and noting male-to-male transmission from I.1 to II.5, and then attributed them to pseudodominance from carrier spouses, concluding autosomal recessive inheritance because SCD is "molecularly known to be an autosomal recessive hemoglobinopathy" (Supplementary Figure S18). ChatGPT concluded autosomal recessive inheritance, stating that SCD "is inherited in an autosomal recessive pattern" and that "the pedigree perfectly matches" (Supplementary Figure S5). Gemini reported that the image did not load and answered autosomal recessive on the basis of "typical presentations for this condition" (Supplementary Figure S9). Copilot identified autosomal dominant inheritance from vertical and male-to-male transmission but described the condition only as "a sickling hemoglobinopathy" without explaining how it could be dominantly inherited (Supplementary Figure S14).

## Discussion

This study compares the performance of four consumer AI assistants, ChatGPT, Gemini, Claude, and Copilot, on image-based tasks involving pedigree analysis pertinent to undergraduate medical education in genetics. In this task set, ChatGPT and Claude obtained the highest accuracy; however, with 19 sub-questions, a single attempt per model, and no statistical testing, these differences are descriptive and should not be interpreted as an established ranking. Copilot showed intermediate performance, and Gemini showed poor performance, largely because it repeatedly reported that it could not see, load, or read the pedigree images. These findings demonstrate variability in the performance of consumer AI assistants on visually presented medical genetics tasks. This observation is consistent with previous studies showing that, although modern LLMs perform well on many medical knowledge tasks, visual reasoning and image interpretation remain important limitations for some models, particularly in specialized domains requiring multimodal analysis [24-26]. In the present study, this limitation was particularly evident for Gemini. One of the most important results of the current study is that the ability to succeed in a task involving pedigree-based genetics relies not only on general knowledge of medical genetics but also on proper visual perception of the pedigree structure. This was particularly so in the case of Gemini, whose low final mark followed mainly from its reported inability to see or read the pedigree images. According to the design of this study, those failures were counted as wrong because the aim was to test the real-world performance of consumer-facing tools as students would use them in real-life scenarios. The retrospective error classification is consistent with this interpretation: most of Gemini's incorrect answers followed a reported inability to see or read the image, and Claude's errors in the double-cousin pedigrees arose from misreading the pedigree lines rather than from incorrect knowledge of DNA sharing between relatives.

ChatGPT was most effective for standard pedigree questions, such as recognizing patterns of inheritance and inferring genotypes, but performed poorly on complex DNA-sharing questions. In the authors' assessment, its replies were generally comprehensive and well explained. Additionally, ChatGPT made fewer perception/structure errors than Claude in the double-cousin pedigrees, and its explanations generally set out its reasoning step by step. Such explanations may be useful to learners as a supplementary resource, but this study did not measure learning outcomes, and transparent reasoning was not always correct; for example, ChatGPT assigned double first cousins to the second degree of relationship on the basis of their expected DNA sharing. Nonetheless, good performance on the common trends must not be taken as a comprehensive reliability when it comes to rare or exceptional cases. This was manifested in one pedigree, which concerned a dominant sickle syndrome. SCD is known to be an autosomal recessive disease, but in very rare occurrences, there is an autosomal dominant type. This is the type of SCD referred to as HbS-Oman [23]. Mutations in HbS-Oman are two in a single β-globin allele, which results in super-polymerization of hemoglobin S (HbS) despite the presence of normal adult HbA [23]. The pattern could not be identified by ChatGPT, although it reached the correct diagnosis; however, in the pedigree analysis, it was diagnosed as autosomal recessive when the pedigree was clearly autosomal dominant. Moreover, it was not able to explain the scenario.

Although Claude and ChatGPT demonstrated similar accuracy levels, Claude responses were more detailed, sometimes providing information beyond the scope of the expected answer. Its main observed weakness was the misreading of complex pedigree structures. This was evident in the third pedigree, which showcased a double cousin pattern (the individuals in the third generation are first cousins from both paternal and maternal sides). In this case, Claude misinterpreted the pedigree structure entirely, identifying the individuals as full siblings (50% DNA shared and first-degree relatives) rather than double first cousins. Because Claude's responses explicitly treated III.1 and III.2 as children of the same couple, these errors were classified as perception/structure errors rather than reasoning/knowledge errors (Supplementary Figures S15-S16). This shows that, while sound and detailed knowledge of medical genetics is important, visual accuracy also plays a substantial role in correct analysis of pedigree structures.

Copilot demonstrated average performance in general and responded to a number of traditional questions on inheritance correctly; however, its weaknesses were more evident when it had to interpret accurate information with particular attention to minor details of the pedigree. This was most apparent in the two-cousin pedigrees, in which appropriate computation of shared DNA required the realization that the structure of the relationship was more complicated than that of regular first cousins. In one case, Copilot's answer (12.5%) corresponded to the DNA sharing expected for ordinary first cousins, although its answer-only response did not show whether this reflected misreading of the pedigree or misapplication of the relationship rule. In another, it reached the correct numerical percentage only by reading III.1 and III.2 as full siblings and therefore failed to offer a biologically plausible explanation for why such a number was relevant. These results suggest that, in this task set, Copilot performed adequately on simple pedigree-related recognition tasks but was less reliable with pedigrees that have unusual or neglected architecture.

The special pedigree case of the dominant sickle syndrome scenario was the other case that all four assistants had problems with. Although this was a single case, it illustrates that even the higher-scoring systems can fail when the pedigree is characterized by a rare or unusual manifestation of genetics instead of a typical textbook picture. It suggests that success on common inheritance questions does not necessarily extend to atypical presentations, although a single pedigree cannot establish how these models perform with rare conditions in general. It also points to a potential drawback of the application of consumer AI tools to the field of genetics education without a strict approach to supervision. In outlying cases, students might be given answers that seem knowledgeable but are incomplete, simplified, or wrong.

The recorded responses to Pedigree 9 suggest a plausible explanation for this failure. SCD is conventionally taught as an autosomal recessive disorder, and in several responses, this familiar association appears to have outweighed or reshaped the dominant features shown in the pedigree. The clearest example is Claude, which acknowledged that the pedigree "looks like it could be dominant" and identified male-to-male transmission, but then reinterpreted the pattern as pseudodominance by assuming that the unaffected spouses were carriers, explicitly because SCD is "molecularly known to be an autosomal recessive hemoglobinopathy" (Supplementary Figure S18). ChatGPT likewise justified autosomal recessive inheritance by stating that SCD "is inherited in an autosomal recessive pattern" (Supplementary Figure S5); however, because the image it received cannot be verified, its response is not used here as evidence of how the Pedigree 9 image was read. Gemini, which reported that the image did not load, gave the same recessive answer from the clinical description alone (Supplementary Figure S9), showing the answer suggested by the text when no pedigree evidence is used. Conversely, Copilot read the dominant pattern correctly but did not reconcile it with the diagnosis or identify HbS-Oman (Supplementary Figure S14), indicating that a correct reading of the pedigree was not sufficient without knowledge of the rare variant. These observations are consistent with familiar textual knowledge taking precedence over pedigree evidence, but they are inferred from the outputs for a single pedigree and do not demonstrate an internal mechanism of the models. The order of the questions, in which the diagnosis was requested before the mode of inheritance, may also have encouraged anchoring on the typical inheritance of the named disease. Testing this explanation would require several atypical pedigrees, varied question order, and repeated queries.

For medical educators, this pattern has practical implications. A response that names the correct disease and gives a fluent explanation may still misrepresent the pedigree, and the error may be difficult for a novice to detect because it agrees with what the student has been taught. Educators could ask students to check an AI-generated description of a pedigree (affected individuals, matings, and transmission events) against the figure before accepting its conclusion; use atypical inheritance scenarios, such as HbS-Oman or recessive disorders showing pseudodominance, as deliberate teaching cases; and emphasize that the mode of inheritance should be inferred from the pedigree itself, with textbook associations used to check rather than replace that inference.

These findings have several possible implications for learning, although educational outcomes were not measured in this study. First, the AI assistants used by consumers may be of some use as an addition to the standard pedigree-based activities, especially when they offer well-defined step-by-step guidance. Second, their output must be taken with caution, particularly with image-based inquiries, aberrant family setups, and uncommon genetic processes. Third, wrong but plausible-sounding reasons can be especially problematic in the educational context, as they may be used to reinforce a false assumption rather than correct the misconception. The two error types also call for different safeguards: perception errors can be detected by checking that the description of the pedigree given by the AI matches the figure, whereas reasoning errors require learners to verify the genetic principle being applied. These findings suggest that AI-generated solutions should not substitute for formal instruction, answer keys, and professional guidance in medical genetics education [5,24].

This study has limitations. It was analyzed using a total of nine pedigree cases; however, there were two to three related questions per case. The sample was thus adequate in an exploratory comparative study but limited. The assistants were also given just a single attempt, with no retests, paraphrasing of statements, or additional prompts. Although this method is deliberate and is representative of a realistic consumer-use situation, it might be overly conservative in terms of predicting the performance that might be attained using iterative prompting. Nevertheless, they should not have been provided with multiple retries to test prompt optimization, but first of all, first-pass usability, which was not the objective of this study. The other limitation is that the study was narrow in that it concentrated on consumer-facing versions of the assistants, and therefore, the results cannot be considered generalizable to underlying foundation models and enterprise versions with other capabilities.

Several further limitations should be considered. No statistical testing was performed, so the differences between models are descriptive, and learning outcomes were not measured. All testing took place on a single day using the model versions then available in the free consumer interfaces; because these models are updated frequently, the results may not be reproducible with later versions, and the underlying model of the consumer Copilot app was not documented. Detailed scoring rules for partial, contradictory, and open-ended answers were not recorded at the time of testing and were defined during revision, when the item-level re-check changed two item scores and corrected three totals. The answer key was reviewed by one medical geneticist who is also a coauthor, rather than by multiple independent geneticists; although the key for the HbS-Oman pedigree is consistent with the published description of this variant [23], independent review by several experts would strengthen confidence in the key for rare conditions. The pedigree image displayed with ChatGPT's response to Pedigree 9 (Supplementary Figure S5) does not match that response, and the image actually received could not be verified. Finally, the error-source classification was retrospective, was not performed by blinded or independent raters, had no agreement statistics, and relied on the explanations written by the models, which may not reflect how the answers were generated; answer-only responses could not be classified.

Regardless of these constraints, the study answers a practical and timely question: to what extent do frequently used consumer AI assistants solve medical genetics tasks based on pedigree when used in ways students are most likely to use them? In this task set, performance varied considerably across platforms. ChatGPT and Claude obtained the highest scores, whereas Copilot showed intermediate performance and Gemini performed poorly, largely because of its reported inability to see or read the pedigree images. Combined, these results suggest that consumer AI assistants may provide selective educational support in medical genetics, although at present their performance does not support unsupervised use in the interpretation of pedigrees.

## Conclusions

In this exploratory study of nine pedigree cases, consumer LLMs showed substantial variability in their ability to solve image-based pedigree interpretation problems. ChatGPT and Claude obtained the highest scores, Copilot showed intermediate accuracy, and Gemini was markedly limited by reported failures to see or read the pedigree images; because each model was tested once and no statistical testing was performed, these differences should not be interpreted as an established ranking. Retrospective analysis of the responses indicated that errors arose both from misreading pedigree images and from misapplying genetic principles. In the single atypical pedigree tested, the familiar recessive inheritance of SCD appeared to take precedence over the pedigree evidence in some responses. Successful pedigree analysis therefore requires both accurate visual interpretation and sound genetic reasoning, and the consumer AI assistants evaluated here differed in both.

Although consumer AI assistants may serve as supplementary resources for common pedigree-based genetics problems, their educational benefit was not measured in this study, and they made errors with complex pedigree structures and with the single atypical pedigree tested. Therefore, they should complement, rather than replace, formal medical genetics teaching and expert supervision. Continued evaluation of these rapidly evolving models, including repeated testing, larger and more varied pedigree sets, and studies of learning outcomes, will be essential to define their appropriate role in genetics education.

## Figures and Tables

**Figure 1:**
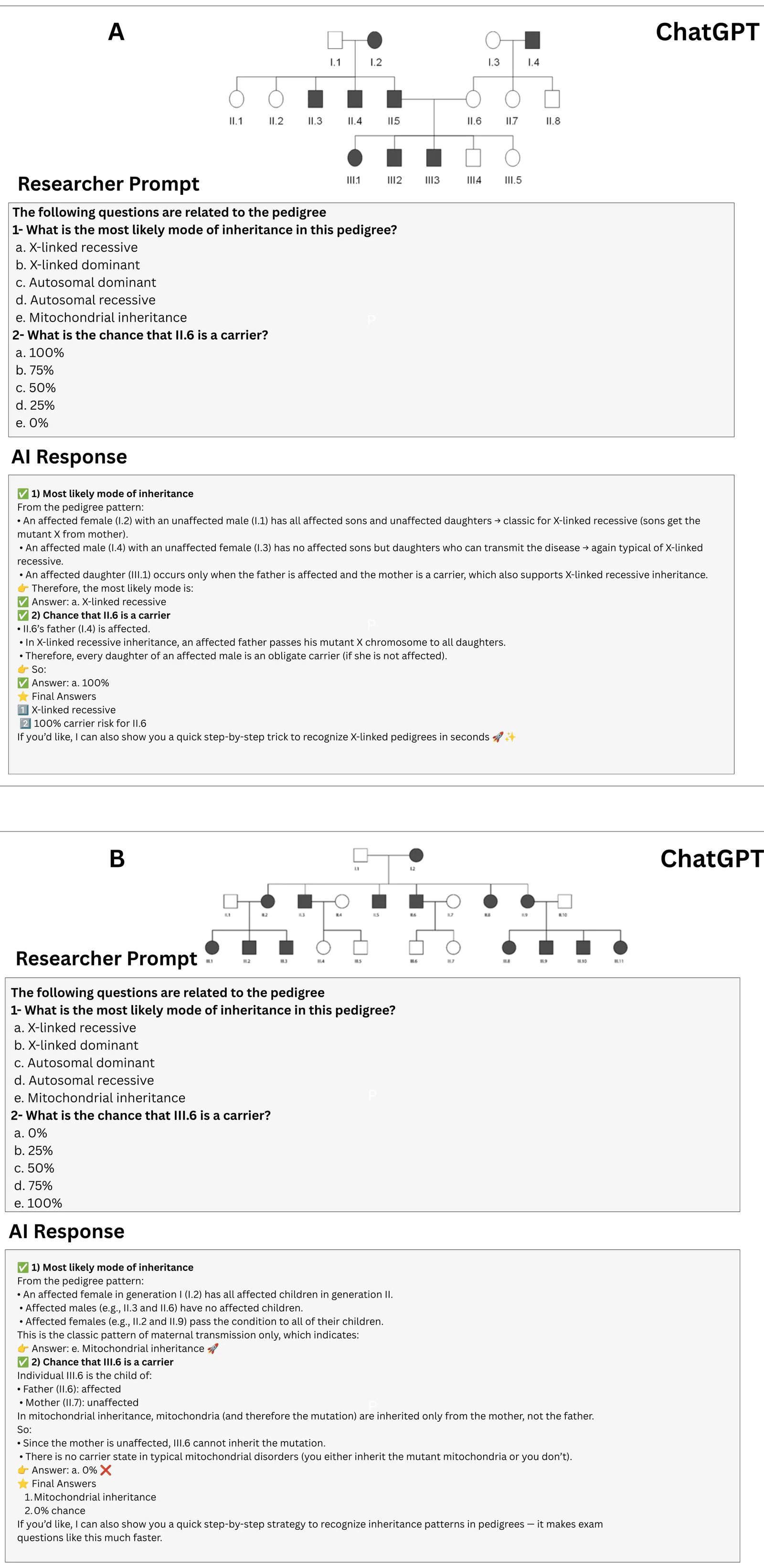
Panel A shows ChatGPT's response to Pedigree 1, and Panel B shows ChatGPT's response to Pedigree 2.

**Figure 2:**
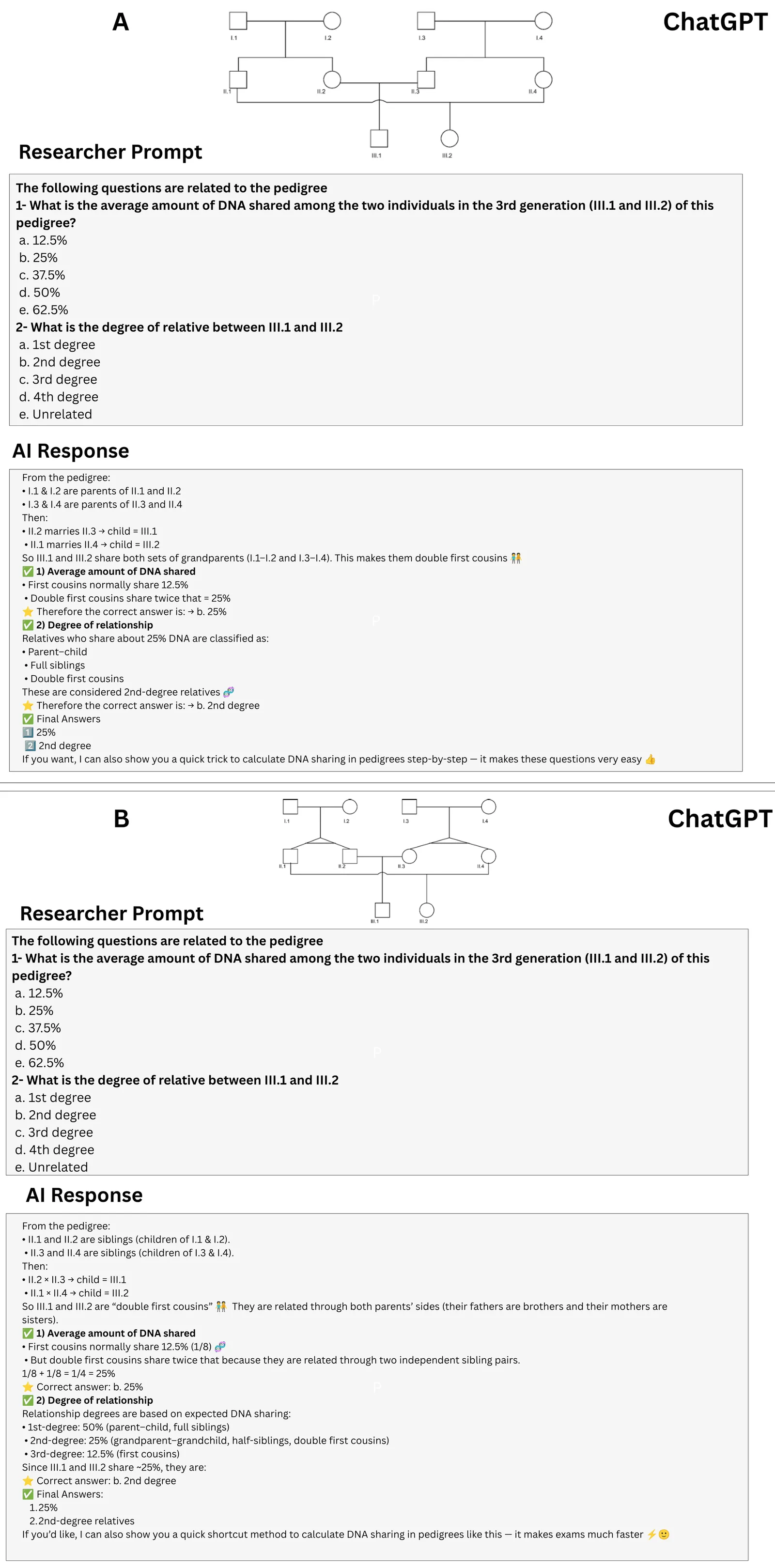
Panel A shows ChatGPT's response to Pedigree 3, and Panel B shows ChatGPT's response to Pedigree 4.

**Figure 3:**
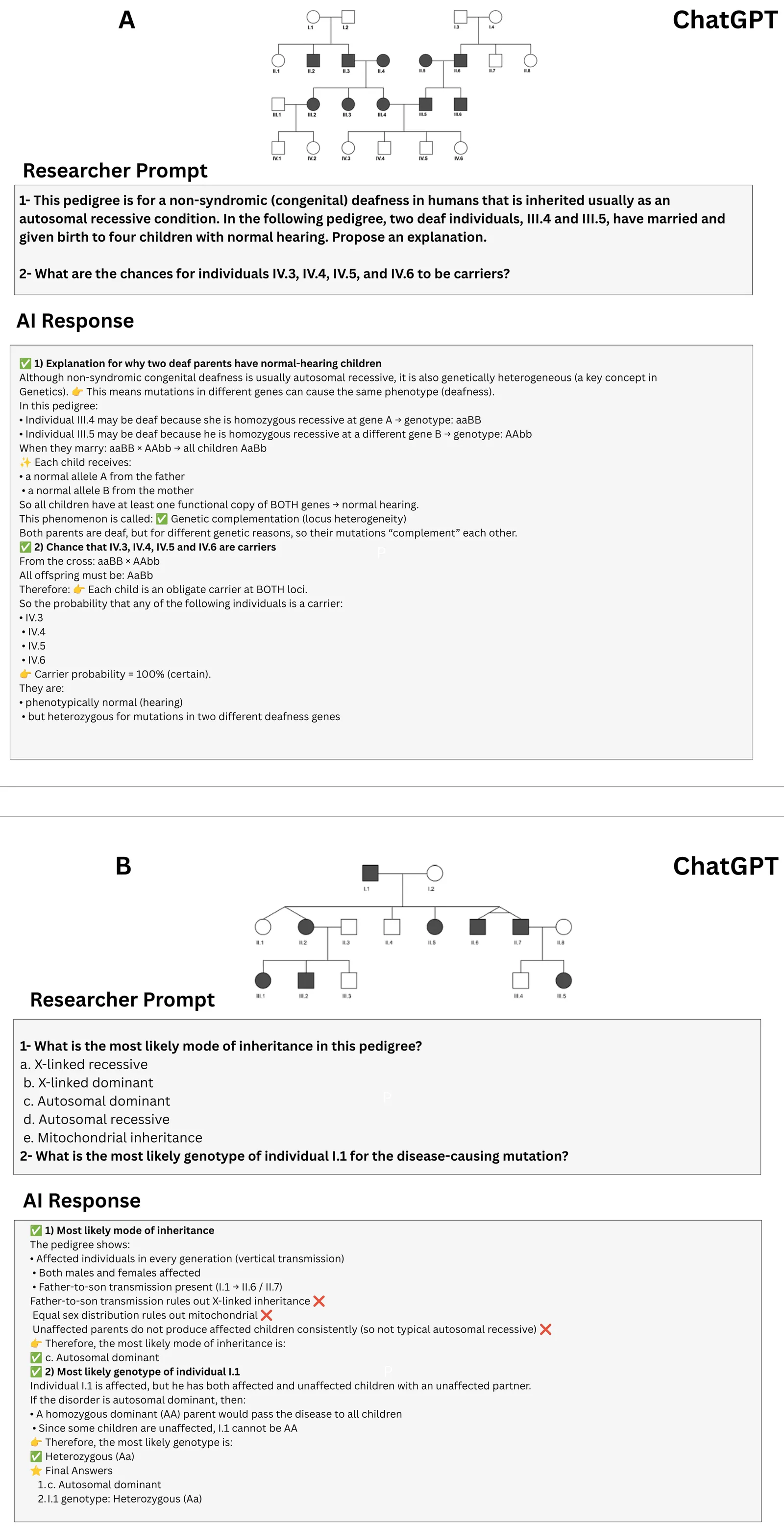
Panel A shows ChatGPT's response to Pedigree 5, and Panel B shows ChatGPT's response to Pedigree 6.

**Figure 4:**
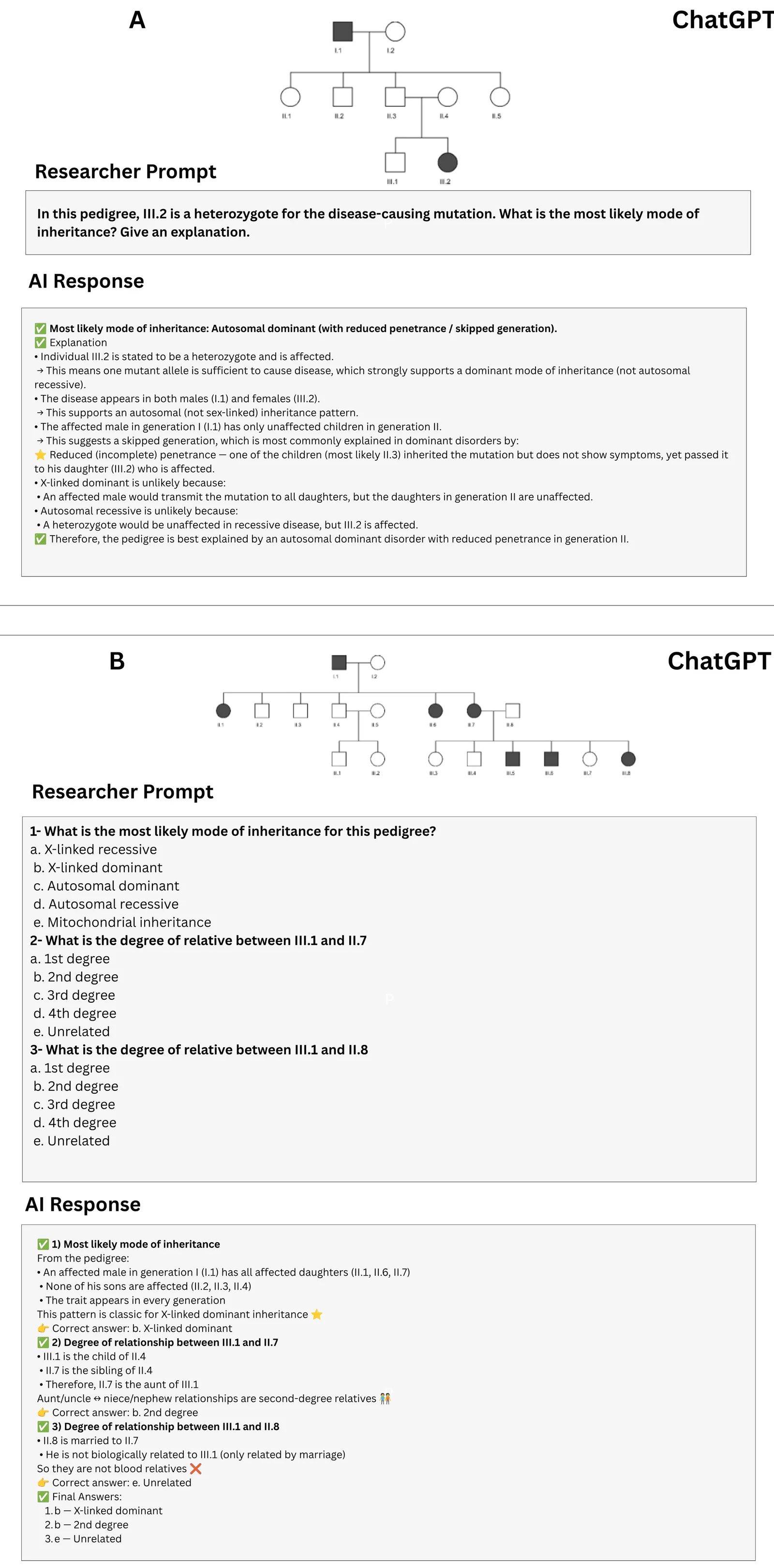
Panel A shows ChatGPT's response to Pedigree 7, and Panel B shows ChatGPT's response to Pedigree 8.

**Figure 5:**
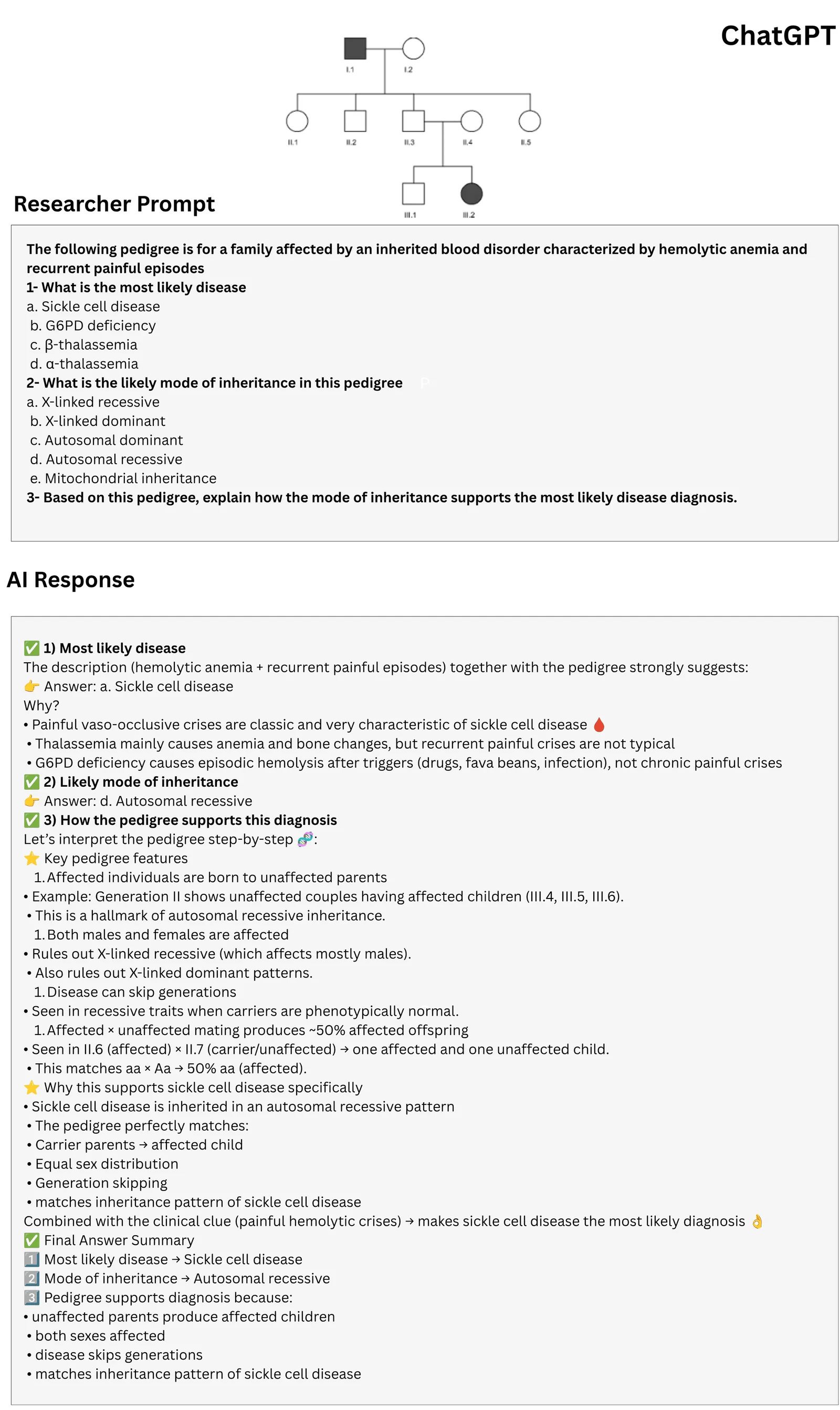
ChatGPTs response to Pedigree 9.

**Figure 6:**
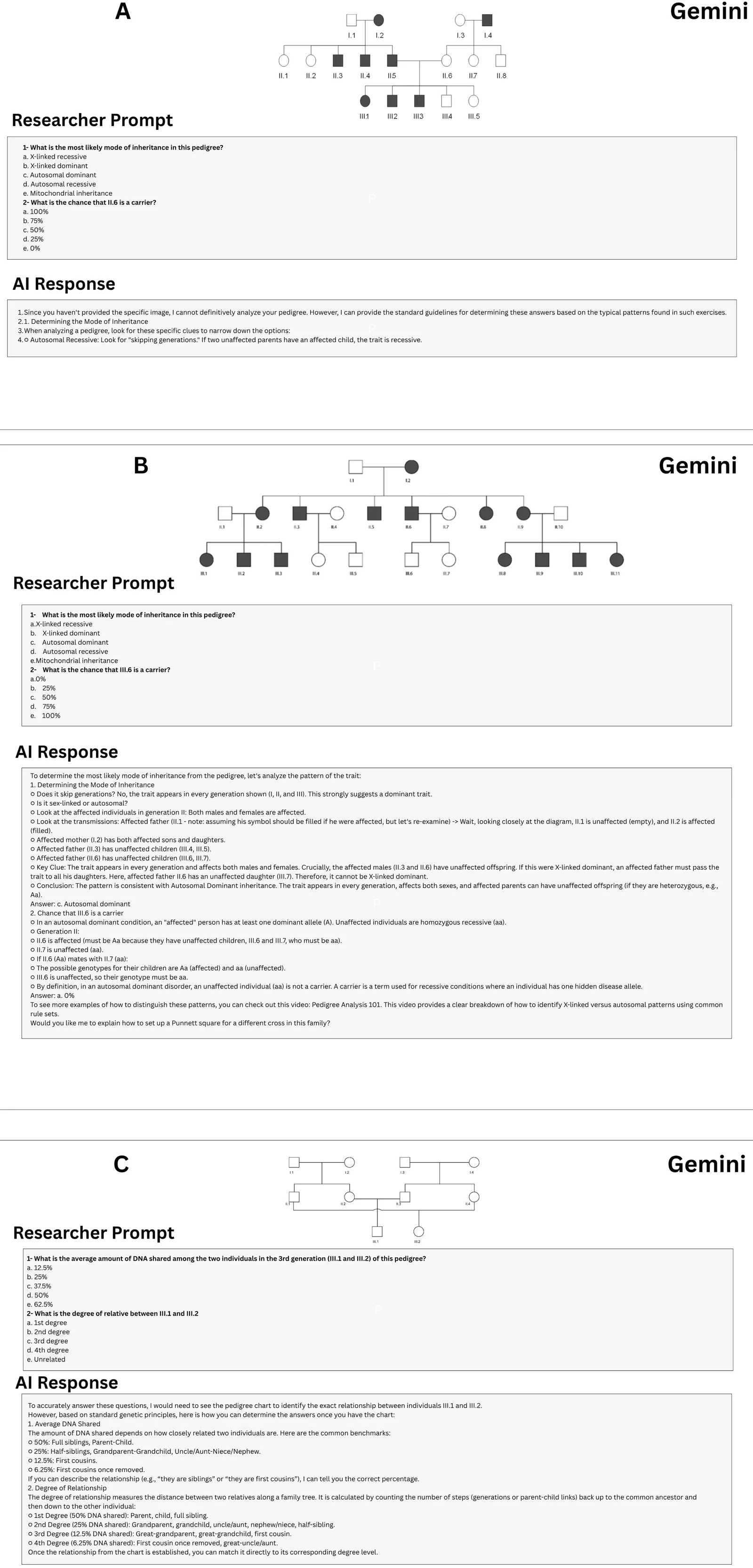
Panel A shows Gemini's response to Pedigree 1, Panel B shows Gemini's response to Pedigree 2, and Panel C shows Gemini's response to Pedigree 3.

**Figure 7:**
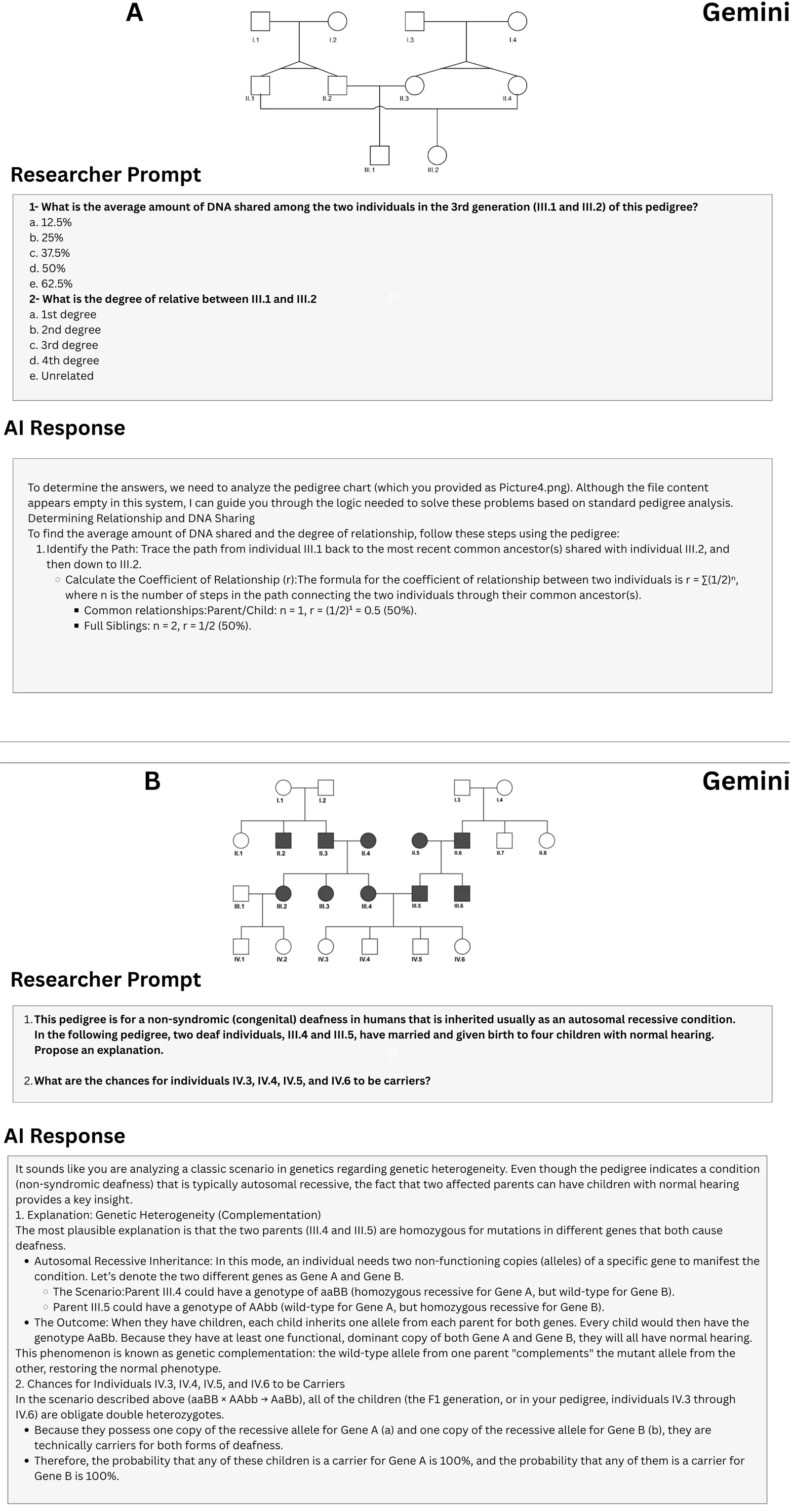
Panel A shows Gemini's response to Pedigree 4, and Panel B shows Gemini's response to Pedigree 5.

**Figure 8:**
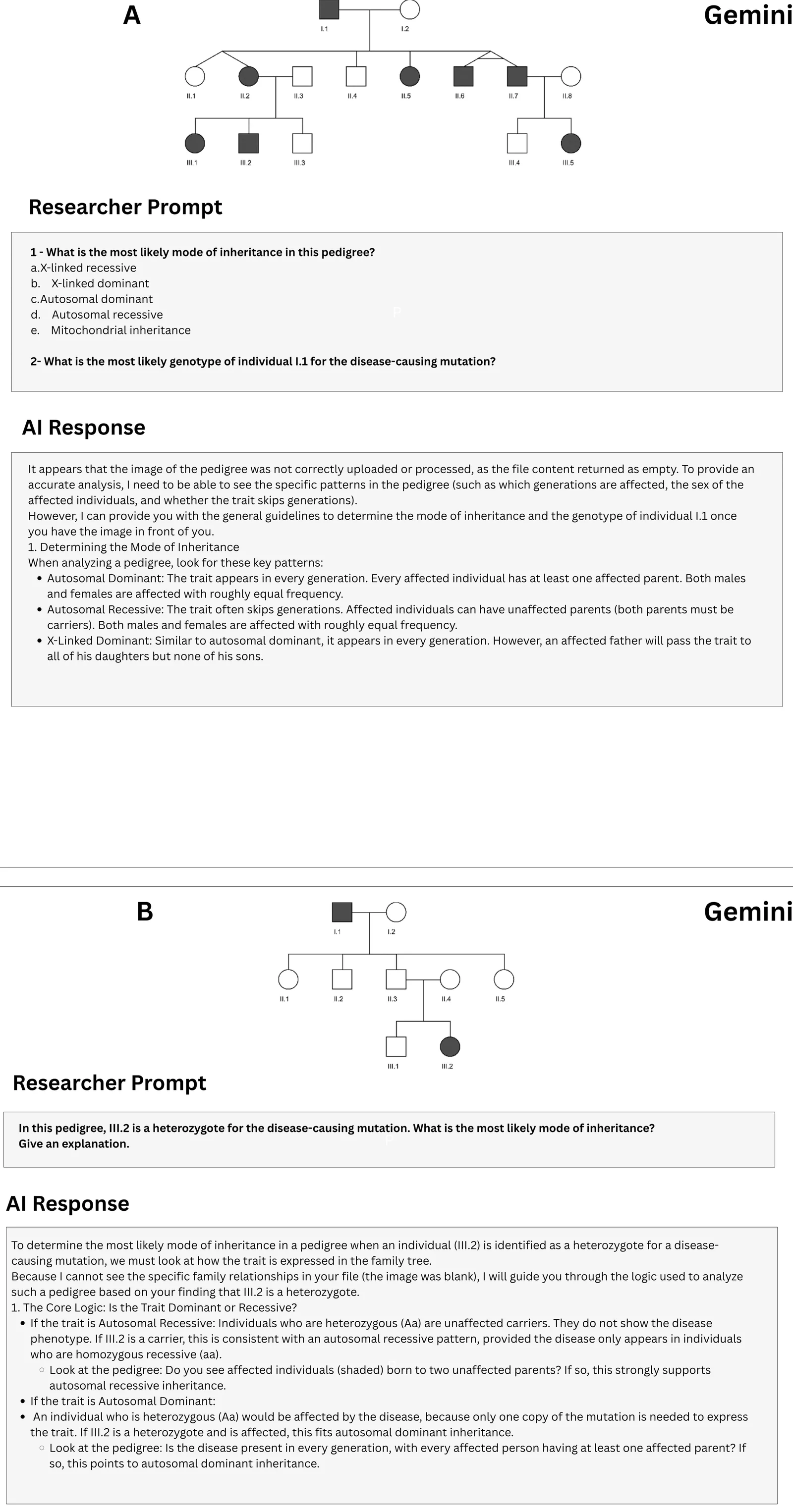
Panel A shows Gemini's response to Pedigree 6, and Panel B shows Gemini's response to Pedigree 7.

**Figure 9:**
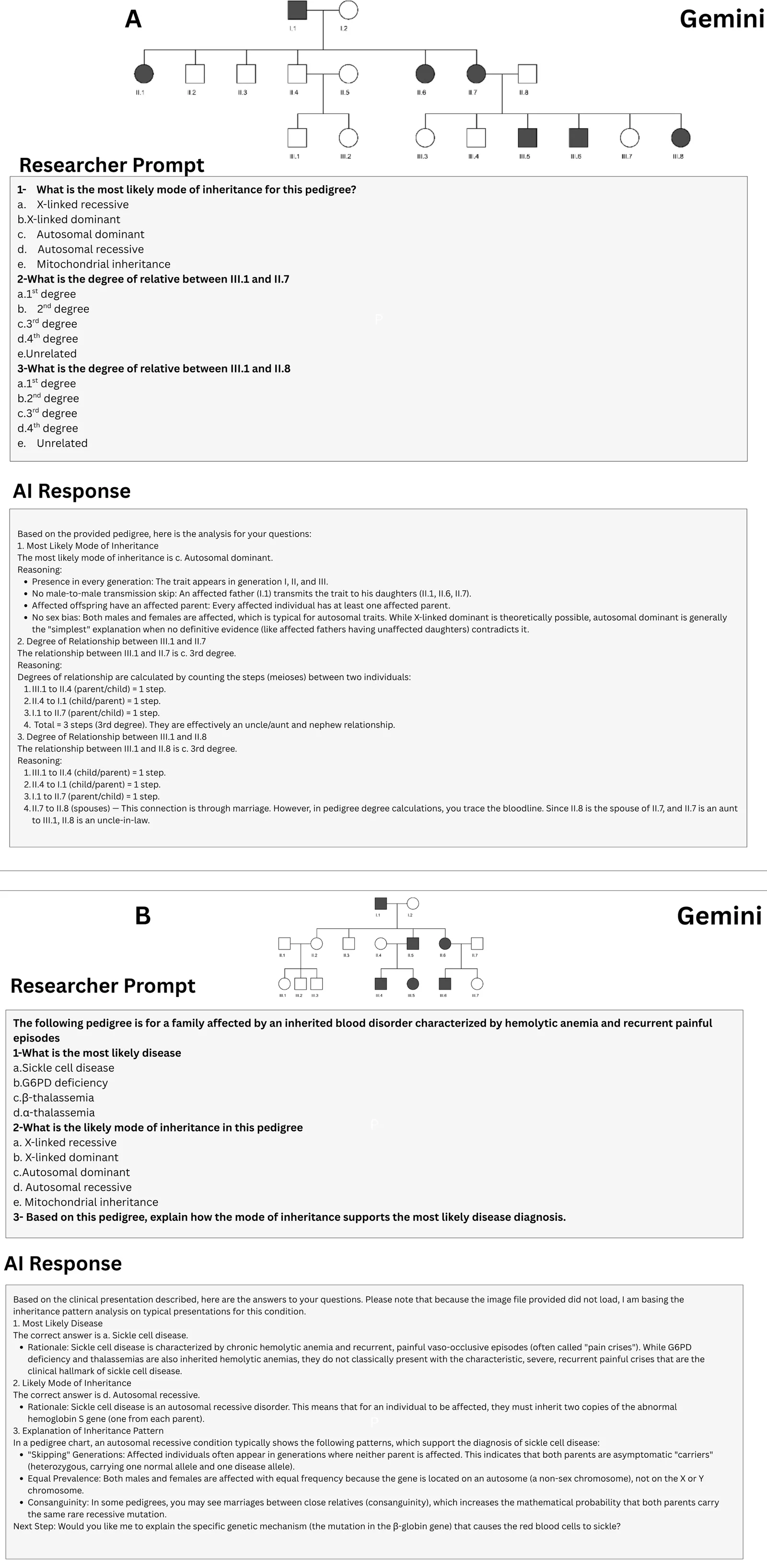
Panel A shows Gemini's response to Pedigree 8, and Panel B shows Gemini's response to Pedigree 9.

**Figure 10:**
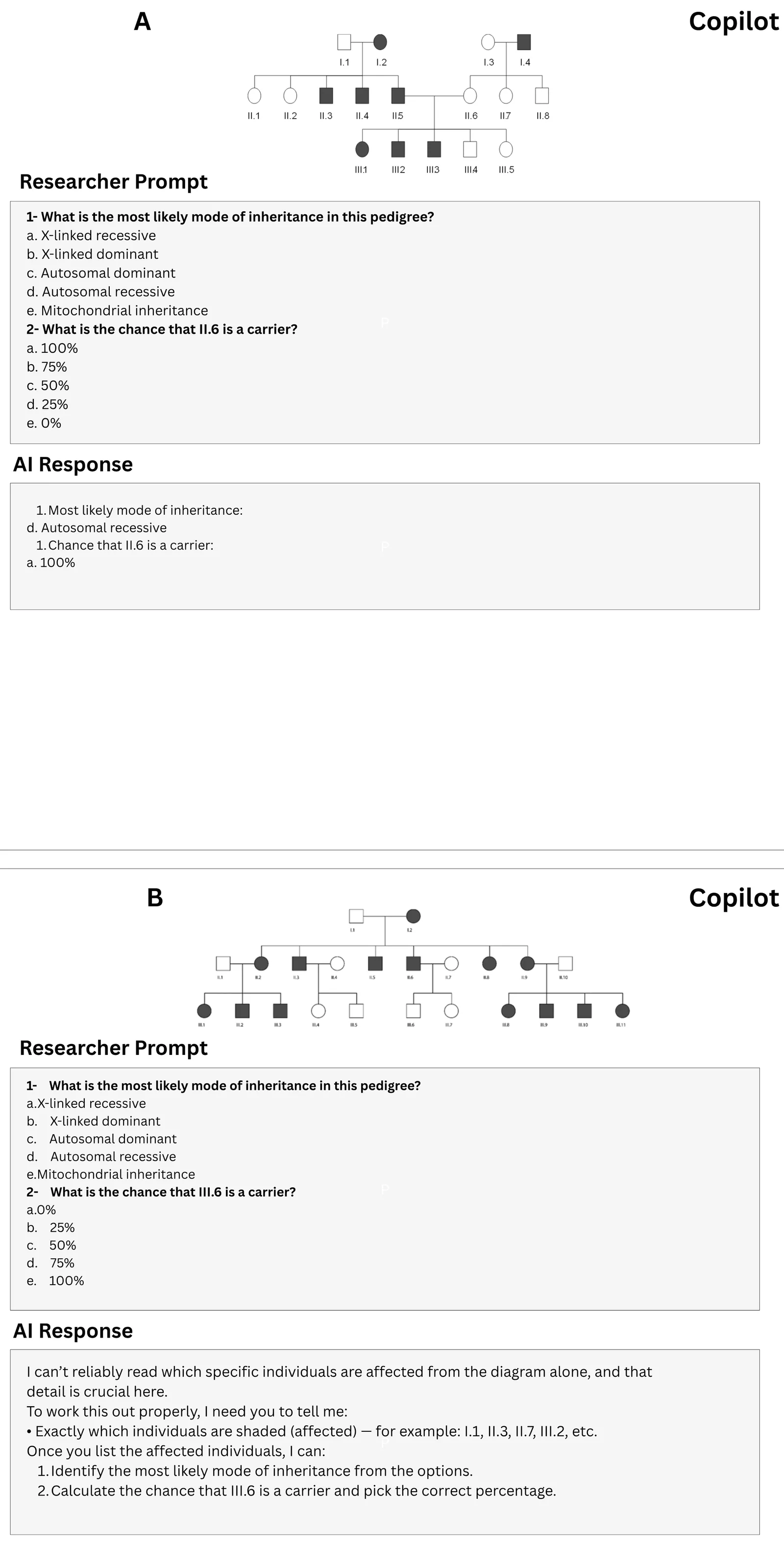
Panel A shows Copilot’s response to Pedigree 1, and Panel B shows Copilot’s response to Pedigree 2.

**Figure 11:**
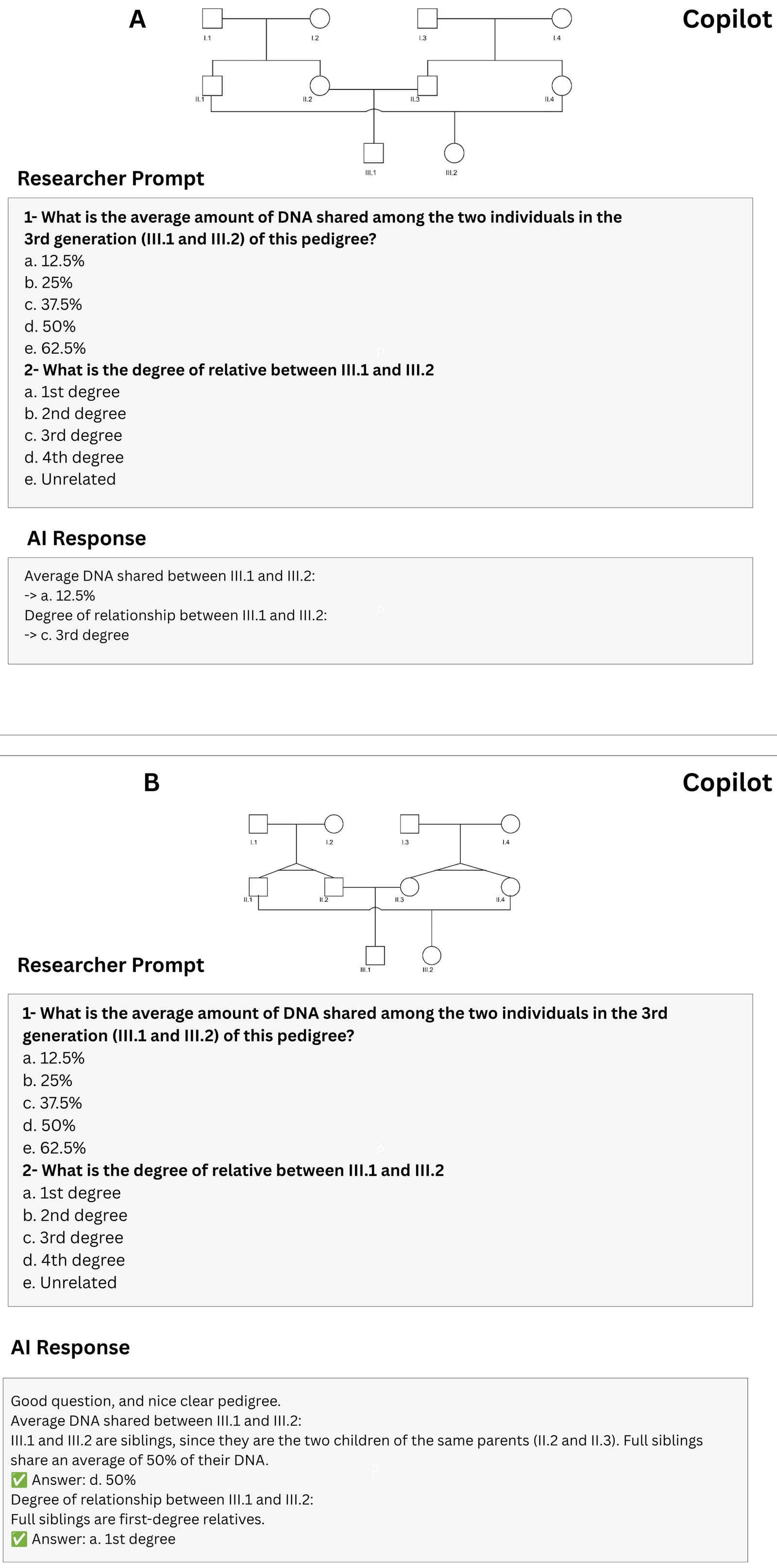
Panel A shows Copilot's response to Pedigree 3, and Panel B shows Copilot's response to Pedigree 4.

**Figure 12:**
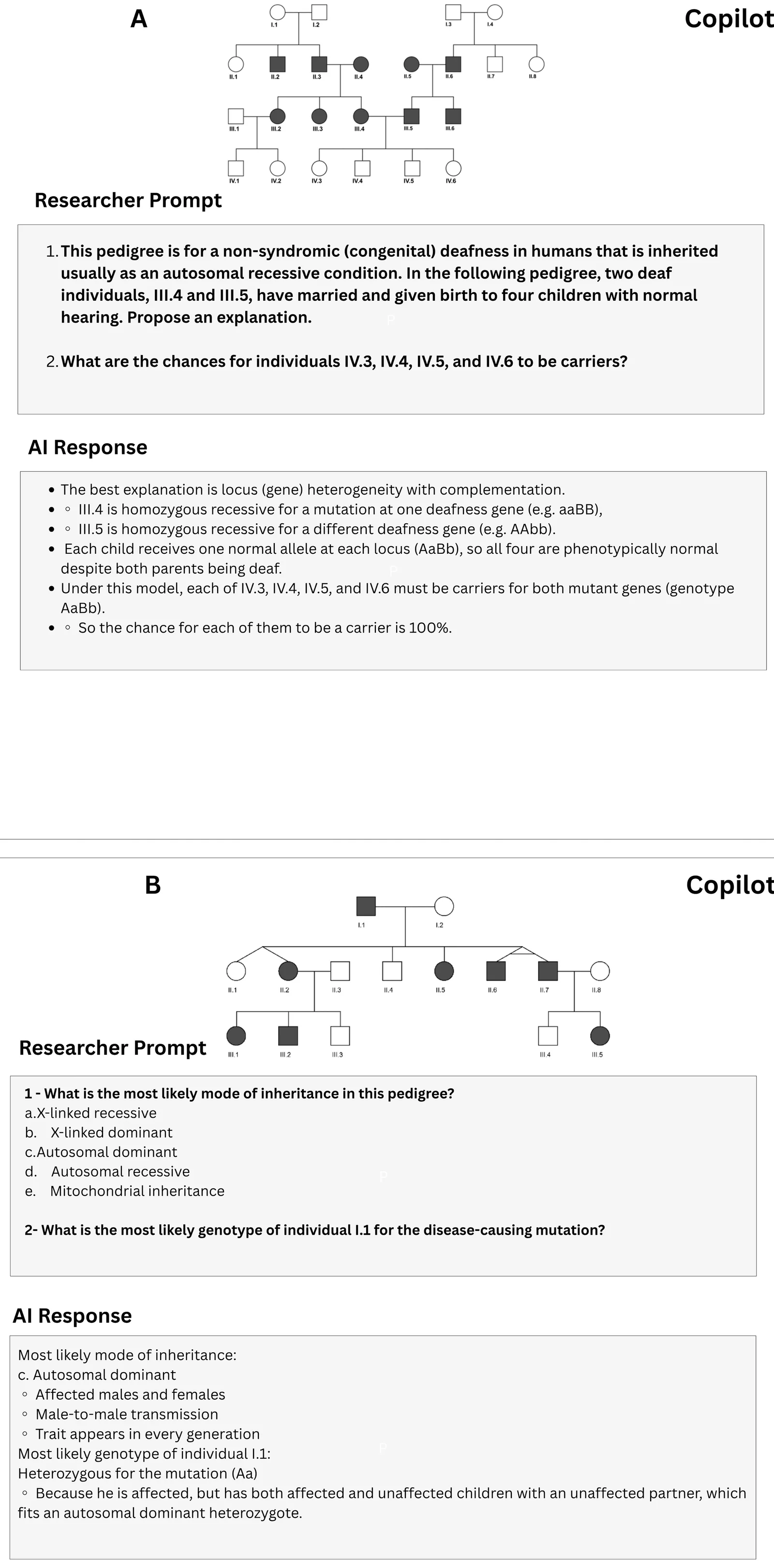
Panel A shows Copilot's response to Pedigree 5, and Panel B shows Copilot's response to Pedigree 6.

**Figure 13:**
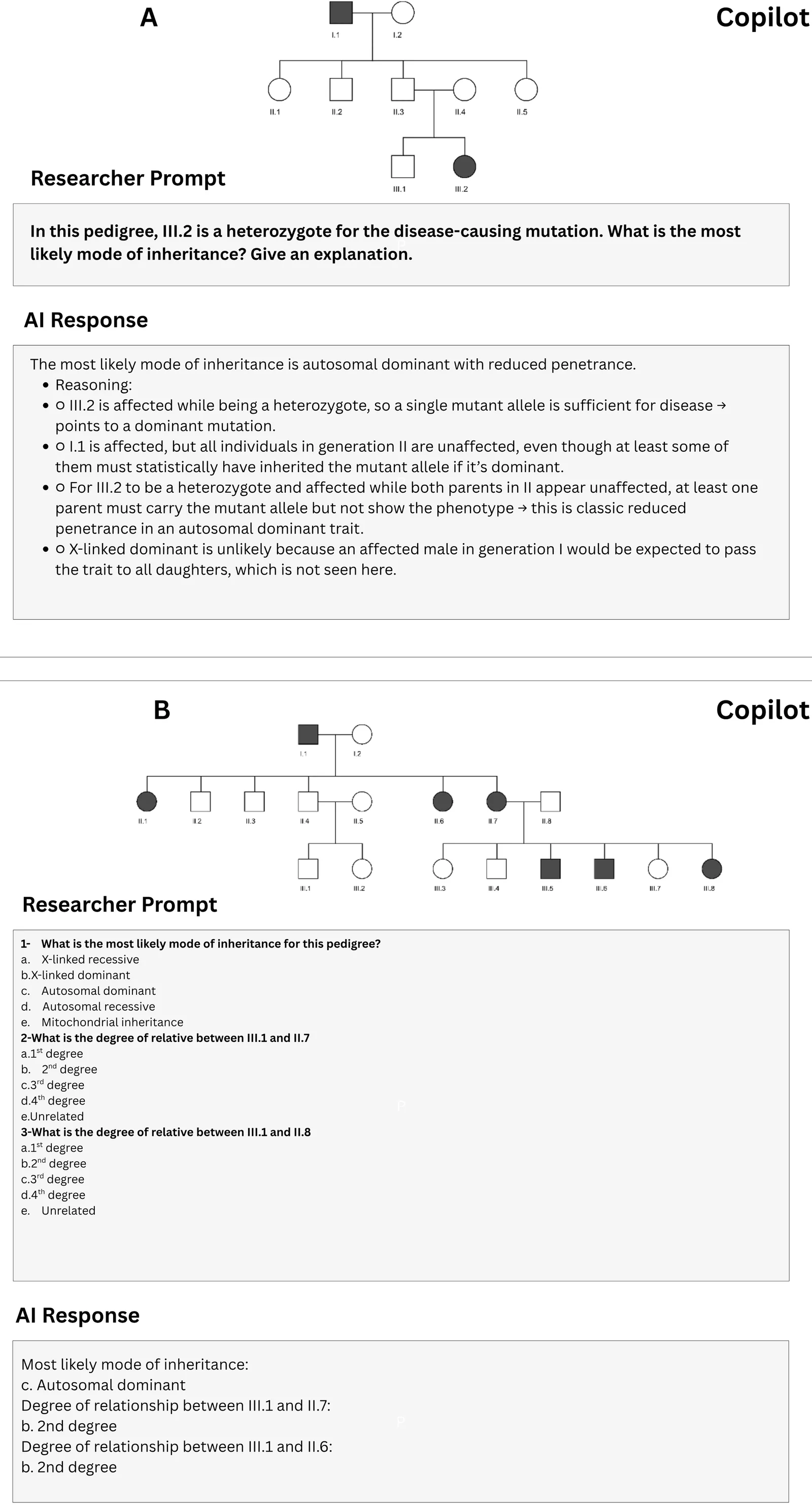
Panel A shows Copilot's response to Pedigree 7, and Panel B shows Copilot's response to Pedigree 8.

**Figure 14:**
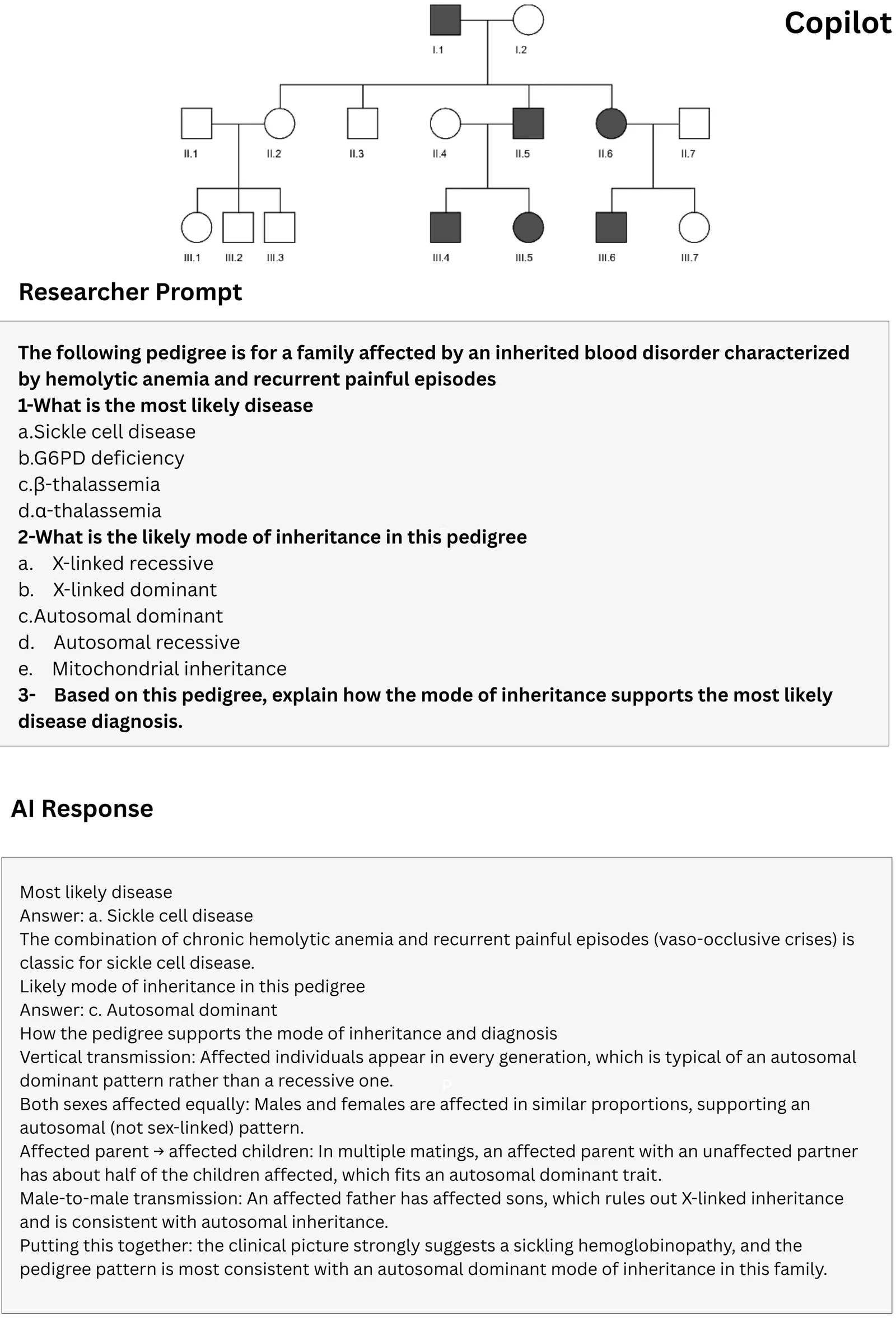
Copilot's response to Pedigree 9.

**Figure 15:**
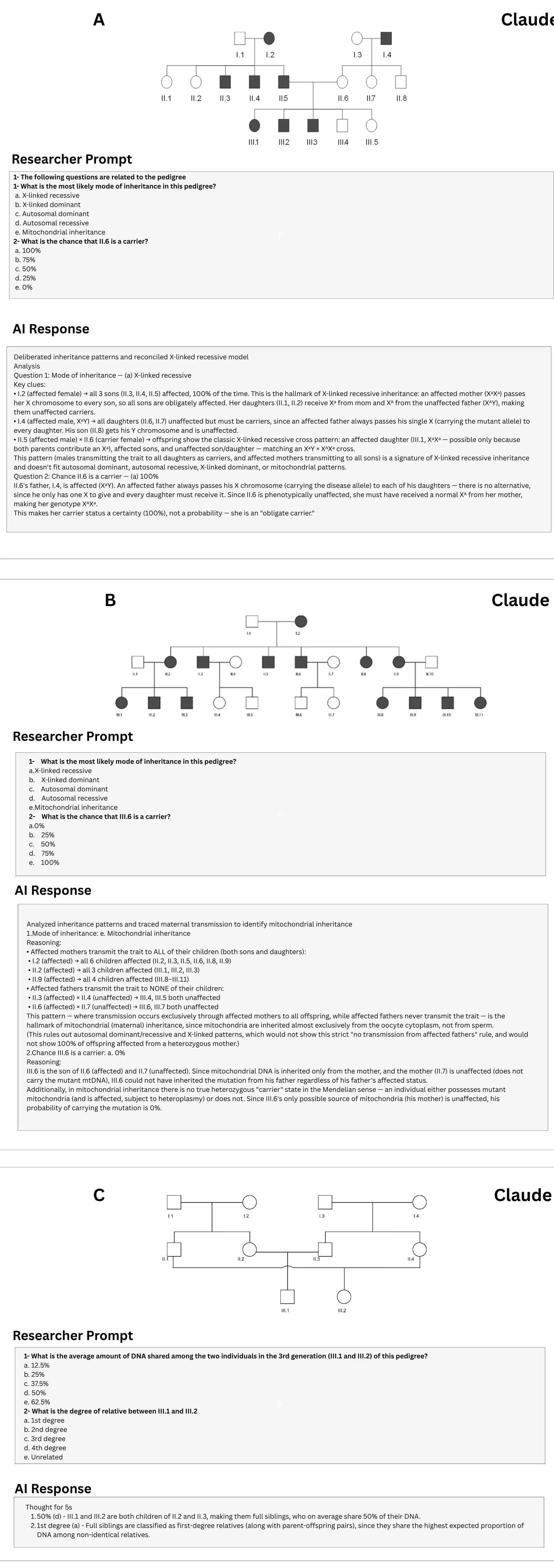
Panel A shows Claude's response to Pedigree 1, Panel B shows Claude's response to Pedigree 2. and Panel C shows Claude's response to Pedigree 3.

**Figure 16:**
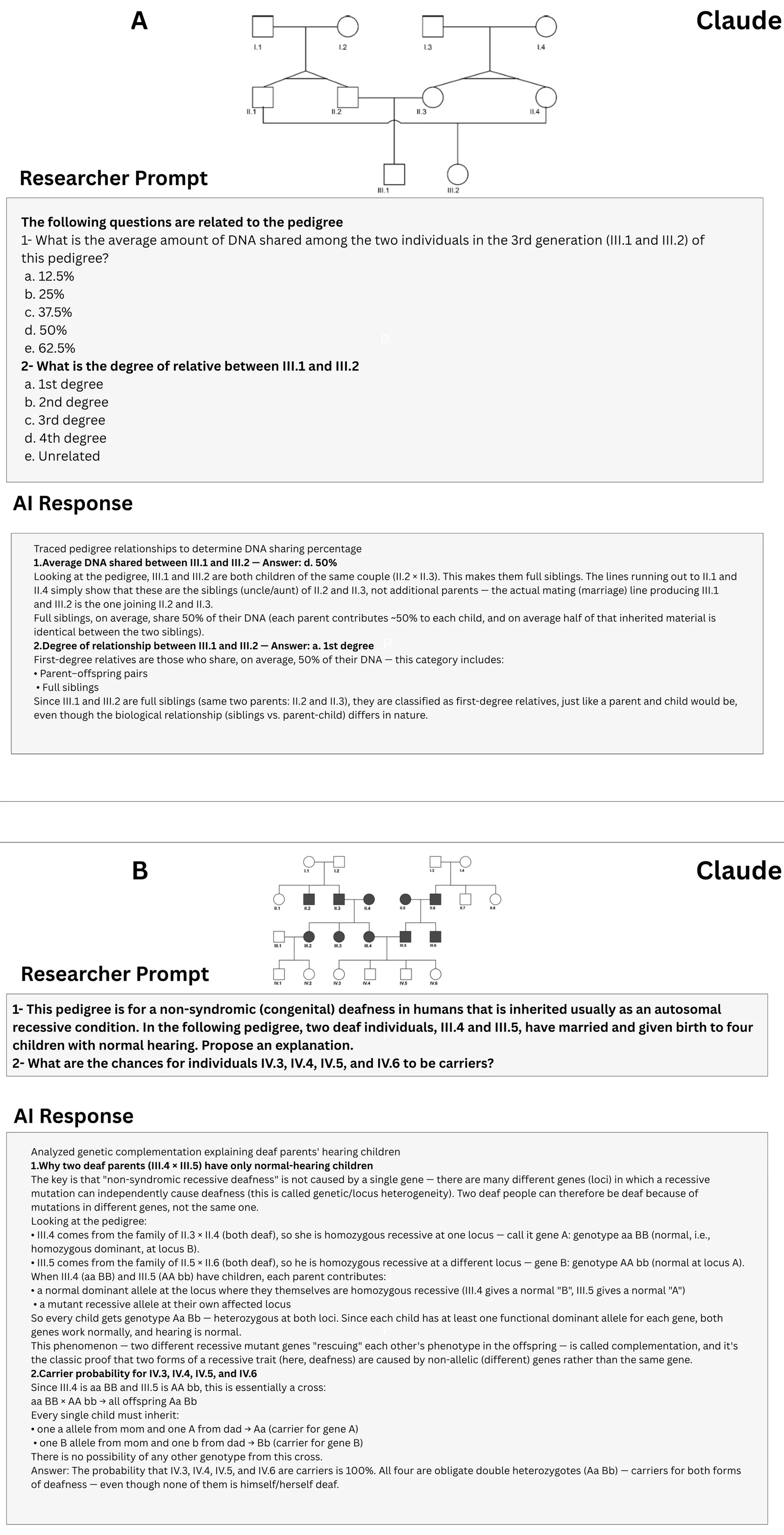
Panel A shows Claude's response to Pedigree 4, and Panel B shows Claude's response to Pedigree 5.

**Figure 17:**
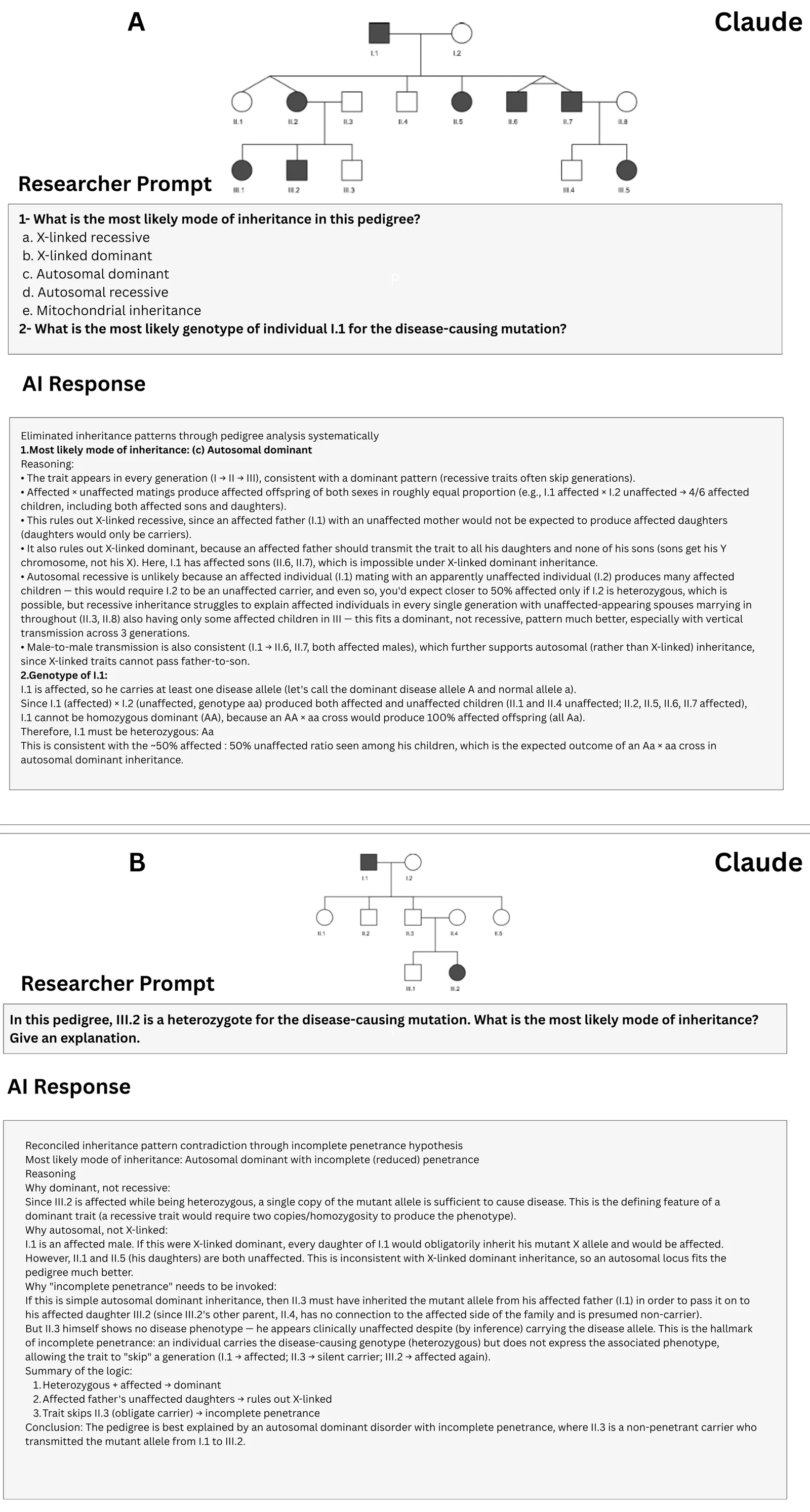
Panel A shows Claude's response to Pedigree 6, and Panel B shows Claude's response to Pedigree 7.

**Figure 18:**
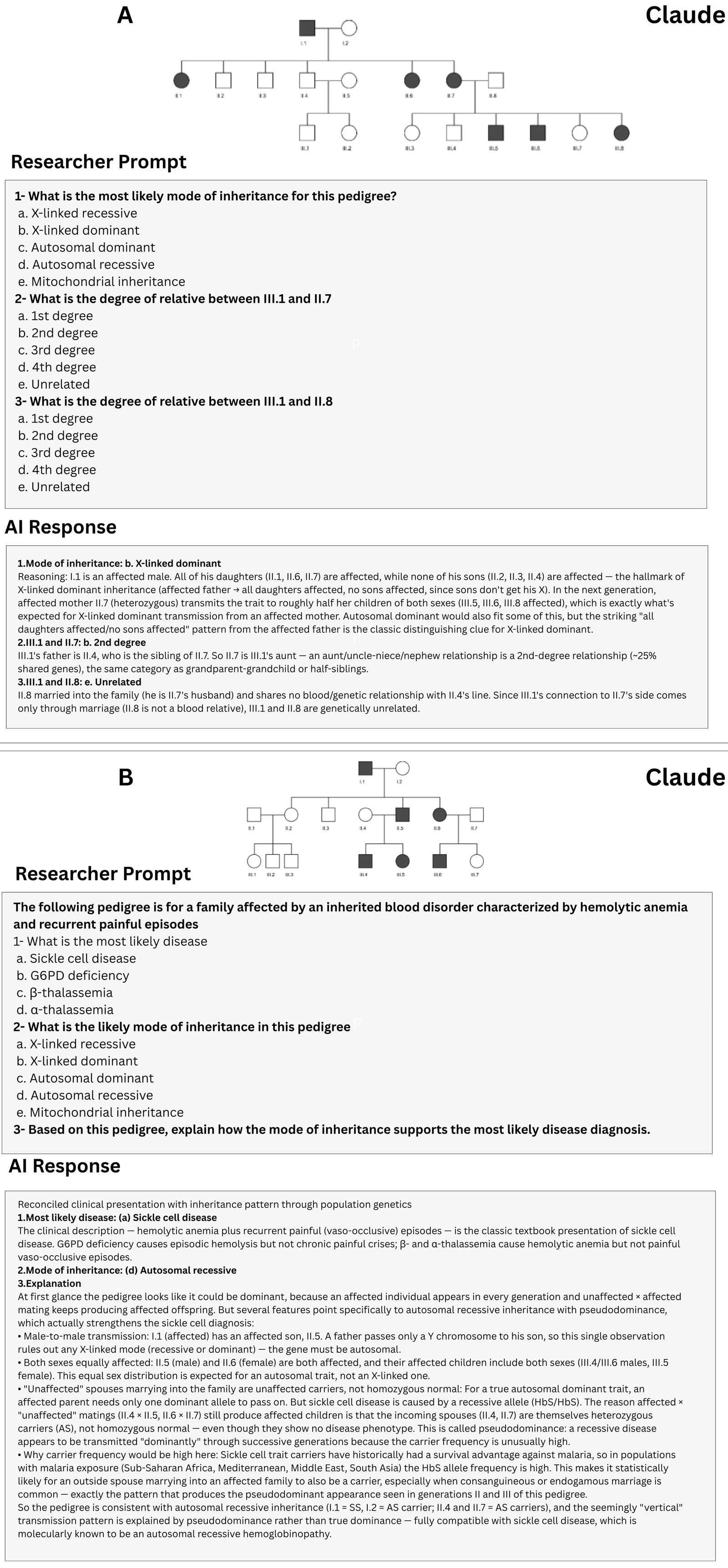
Panel A shows Claude's response to Pedigree 8, and Panel B shows Claude's response to Pedigree 9.

**Figure 19:**
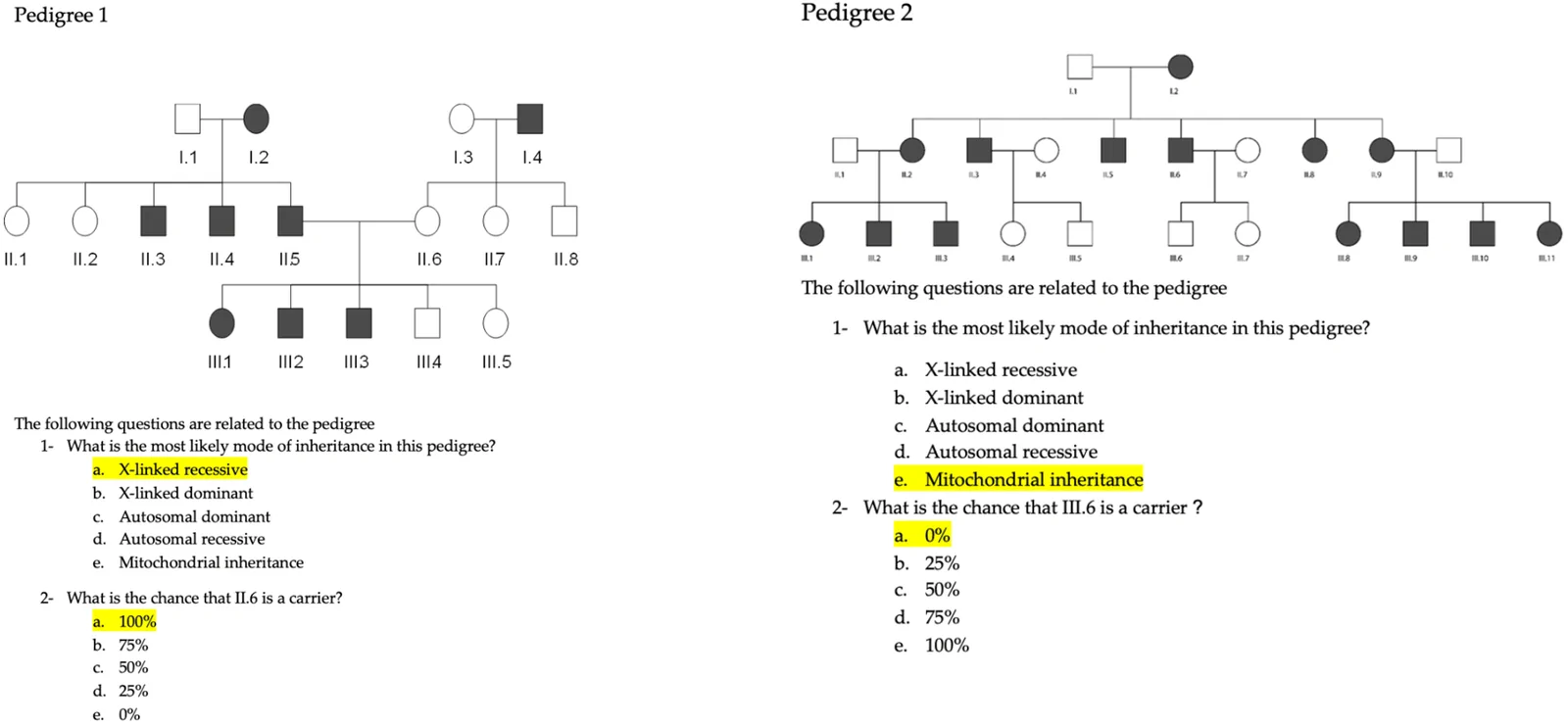
Answer key to Pedigree 1 & 2.

**Figure 20:**
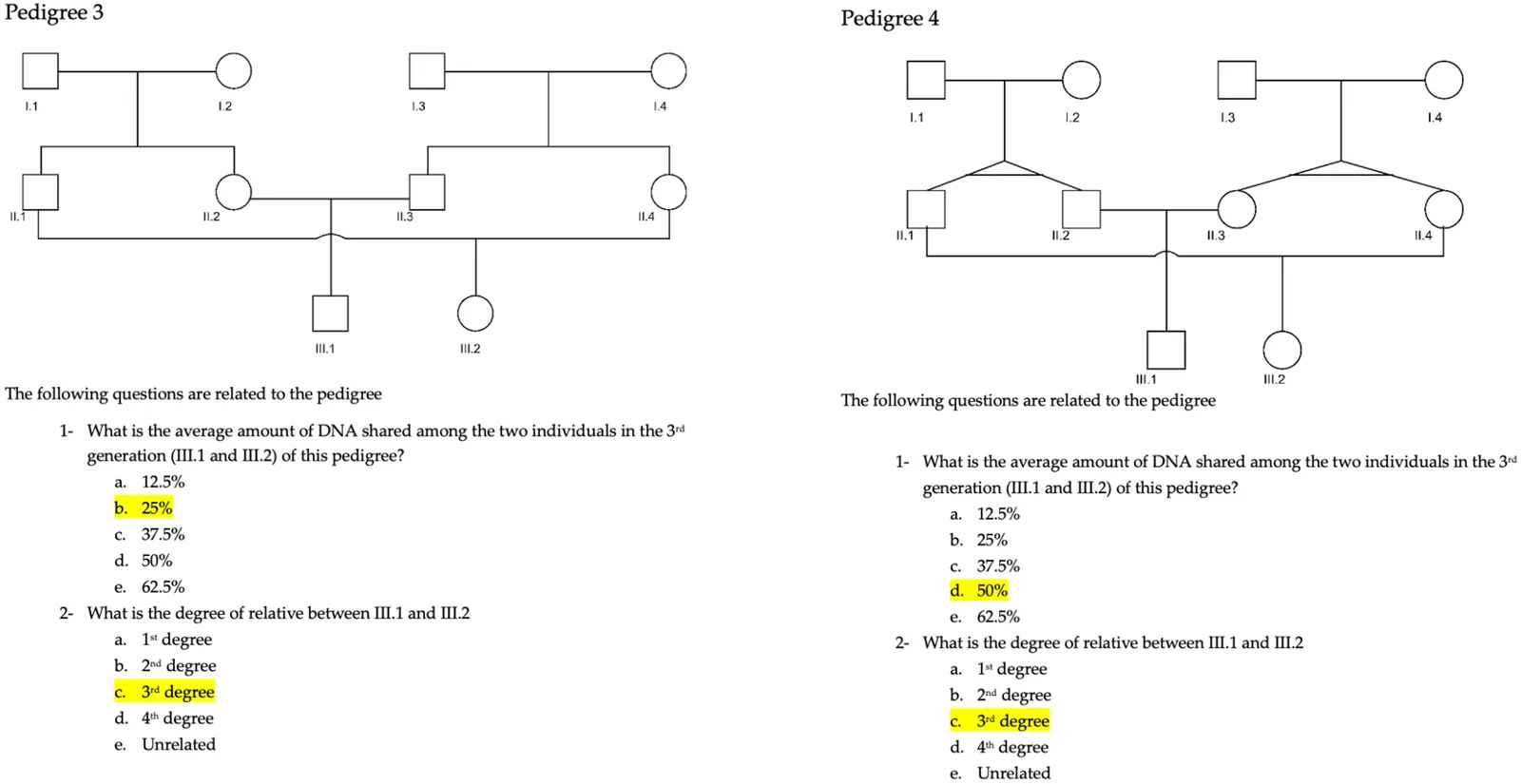
Answer key to Pedigrees 3 & 4.

**Figure 21:**
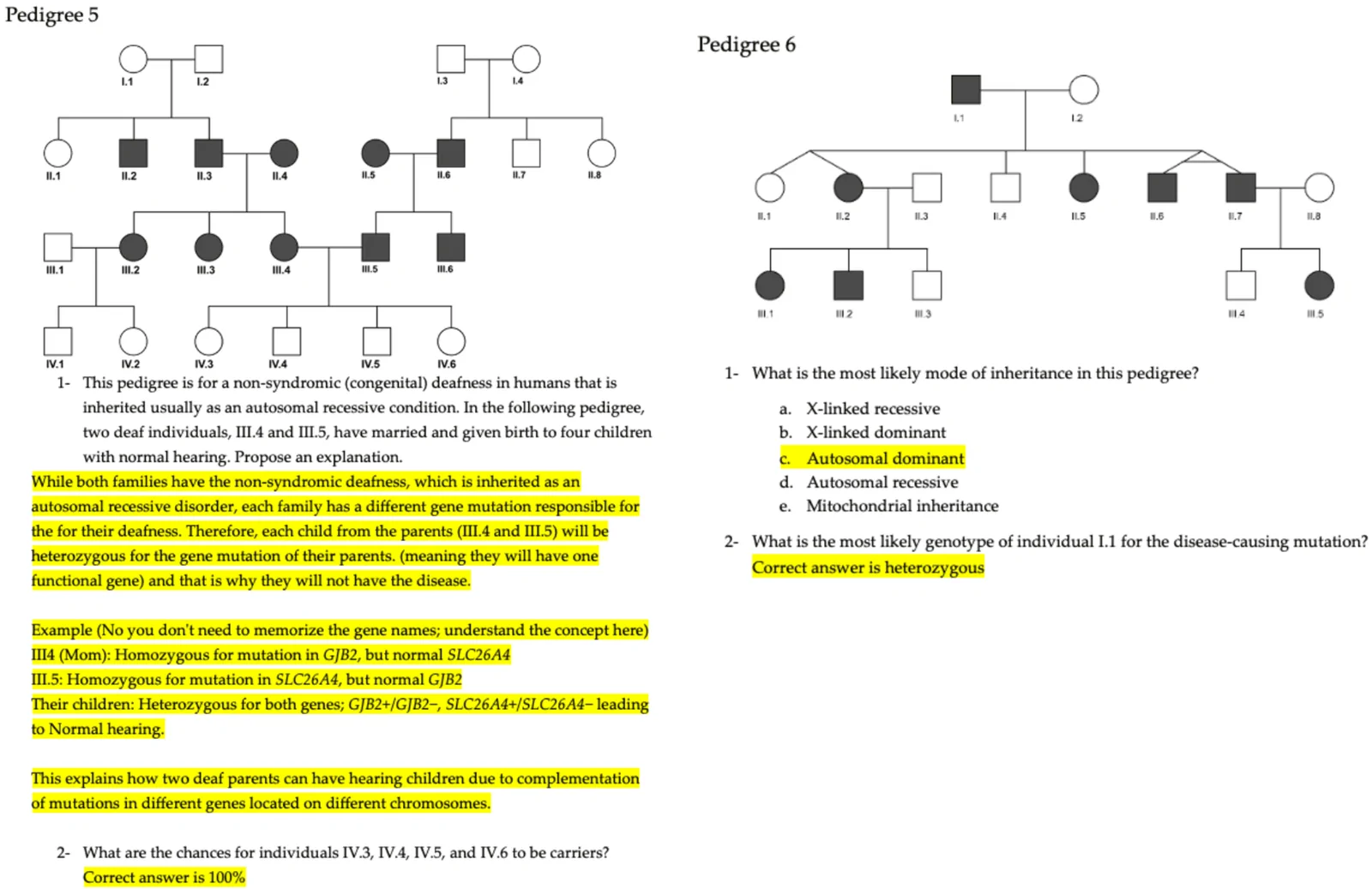
Answer key to Pedigrees 5 & 6.

**Figure 22:**
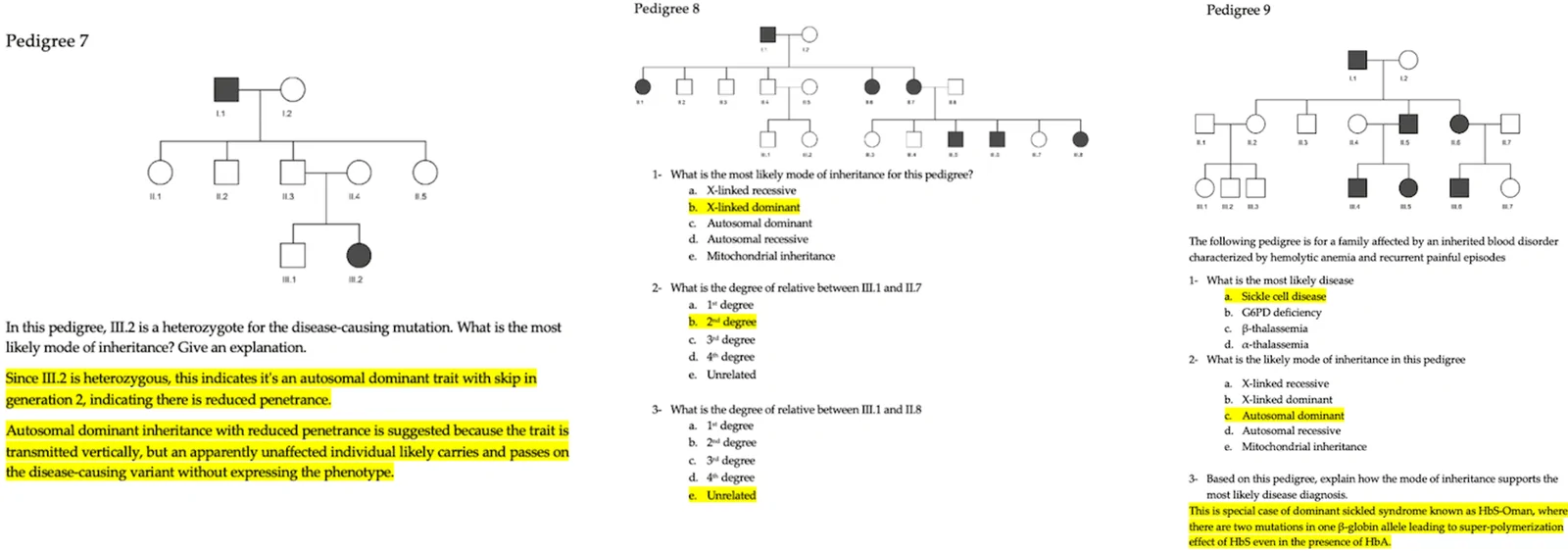
Answer key to Pedigrees 7, 8, and 9.

## Appendices

Appendix 1

Appendix 2
